# Dyrk1b promotes hepatic lipogenesis by bypassing canonical insulin signaling and directly activating mTORC2 in mice

**DOI:** 10.1172/JCI153724

**Published:** 2022-02-01

**Authors:** Neha Bhat, Anand Narayanan, Mohsen Fathzadeh, Mario Kahn, Dongyan Zhang, Leigh Goedeke, Arpita Neogi, Rebecca L. Cardone, Richard G. Kibbey, Carlos Fernandez-Hernando, Henry N. Ginsberg, Dhanpat Jain, Gerald I. Shulman, Arya Mani

**Affiliations:** 1 Cardiovascular Research Center, Department of Internal Medicine, Yale School of Medicine, New Haven, Connecticut, USA.; 2 Department of Pediatrics, Stanford University, Palo Alto, California, USA.; 3 Yale Diabetes Research Center, Departments of Internal Medicine and Cellular and Molecular Physiology, Yale School of Medicine, New Haven, Connecticut, USA.; 4 Department of Comparative Medicine, Yale School of Medicine, New Haven, Connecticut, USA.; 5 Department of Medicine, Vagelos College of Physicians and Surgeons, Columbia University, New York, New York, USA.; 6 Department of Pathology and; 7 Department of Genetics, Yale School of Medicine, New Haven, Connecticut, USA.

**Keywords:** Hepatology, Metabolism, Insulin signaling, Obesity, Protein kinases

## Abstract

Mutations in *Dyrk1b* are associated with metabolic syndrome and nonalcoholic fatty liver disease in humans. Our investigations showed that DYRK1B levels are increased in the liver of patients with nonalcoholic steatohepatitis (NASH) and in mice fed with a high-fat, high-sucrose diet. Increasing Dyrk1b levels in the mouse liver enhanced de novo lipogenesis (DNL), fatty acid uptake, and triacylglycerol secretion and caused NASH and hyperlipidemia. Conversely, knockdown of *Dyrk1b* was protective against high-calorie-induced hepatic steatosis and fibrosis and hyperlipidemia. Mechanistically, Dyrk1b increased DNL by activating mTORC2 in a kinase-independent fashion. Accordingly, the Dyrk1b-induced NASH was fully rescued when mTORC2 was genetically disrupted. The elevated DNL was associated with increased plasma membrane *sn*-1,2-diacylglyerol levels and increased PKCε-mediated IRK^T1150^ phosphorylation, which resulted in impaired activation of hepatic insulin signaling and reduced hepatic glycogen storage. These findings provide insights into the mechanisms that underlie *Dyrk1b*-induced hepatic lipogenesis and hepatic insulin resistance and identify Dyrk1b as a therapeutic target for NASH and insulin resistance in the liver.

## Graphical abstract

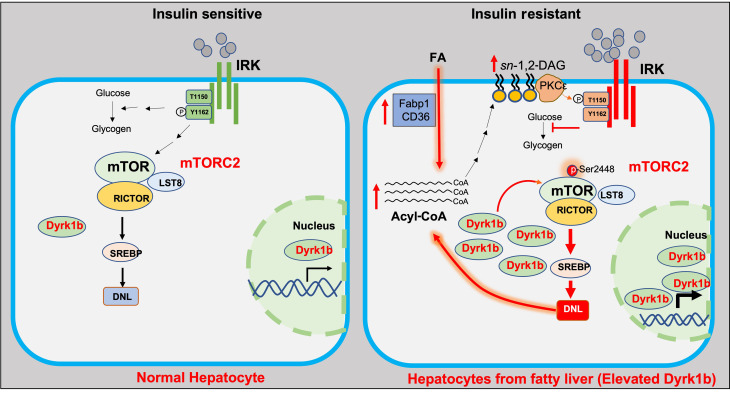

## Introduction

Nonalcoholic fatty liver disease (NAFLD) is a rapidly growing disorder affecting nearly 25% of the adult population worldwide and is a major risk factor for nonalcoholic steatohepatitis (NASH), atherosclerosis, and type 2 diabetes (T2D) (1). The incomplete understanding of the pathophysiology of this disorder has been a major impediment in developing effective therapies. Specifically, pathways that upregulate insulin-dependent hepatic de novo lipogenesis (DNL) in an insulin-resistant liver have remained elusive (2–5).

Under normal conditions, insulin stimulation leads to activation of glycogen synthase causing excess plasma glucose to be stored as glycogen in the liver. The breakdown of glycogen during fasting state maintains glucose homeostasis (6). In an insulin-resistant liver, common in NAFLD and T2D (1), the activation of insulin receptor kinase (IRK) is impaired as a result of increased translocation of PKCε to the plasma membrane, leading to inhibitory phosphorylation of IRK at T1150 residue (7, 8). Reduced hepatic insulin signaling causes decreased activation of glycogen synthase leading to reduced glycogen storage in the liver and increased hepatic glucose production (1, 9).

Concomitant with glucose control, canonical insulin signaling stimulates DNL in the liver, as shown in phosphatidylinositol-3-kinase–deficient (*PI3K-*deficient) mice (5). However, the impaired DNL in this mouse model could not be rescued by overexpression of Akt (RAC-α serine/threonine-protein kinase), suggesting involvement of other downstream effectors in the insulin signaling cascade. Downstream in the insulin signaling pathway, Akt activates mammalian target of rapamycin complex 1 (mTORC1) by directly phosphorylating and inhibiting tuberin in tuberous sclerosis complex 2 (TSC2) (10–12). Paradoxically, hyperactivation of mTORC1 in *TSC1-*knockout liver and reduced activation of the mTORC1/S6K axis were reported to be protective against diet-induced hepatic steatosis and hepatic insulin resistance, respectively, suggesting negative feedback from mTORC1 on insulin signaling (11, 13–15). The glucose-responsive transcription factor carbohydrate-responsive element–binding protein-β (ChREBP-β) has also been shown to transcriptionally regulate the rate-limiting DNL enzymes Fasn and Acc1 and enhance DNL in response to monosaccharides, but its role in glucose metabolism has been conflicting (16). Altogether, these studies are indicative of undiscovered alternate pathways that activate DNL in an insulin-resistant liver.

Most recently, mTOR complex 2 (mTORC2) has emerged as a major driver of lipogenesis in the liver (17–20). The liver-specific knockout of Rictor, an obligate subunit of mTORC2, dramatically reduced lipogenesis (17–19). To date, only a few upstream regulators of mTORC2 have been identified (21, 22), and their relevance to the induction of hepatic steatosis is unresolved. mTORC2 directly phosphorylates Akt at Ser473 residue (23); however, the forced activation of Akt2 failed to drive DNL in mTORC2-deficient mouse liver (17–19).

We had previously reported rare mutations in dual-specificity tyrosine phosphorylation–regulated kinase 1B (*DYRK1B*) that segregated perfectly with the traits of metabolic syndrome (MetS) in several kindreds (24). DYRK1B is a bipartite kinase (25) that is activated by autophosphorylation of tyrosine during translation (26) and phosphorylates its substrates at specific serine/threonine residues (27). The mutations were shown to have kinase-independent gain-of-function effects in increasing autophagic flux (28), and increasing the expression of gluconeogenic and adipogenic enzymes in vitro (24). The role of DYRK1B in regulation of hepatic lipid metabolism in vivo, particularly in the general population, has not been explored.

In this study, we have comprehensively characterized the function of *Dyrk1b* in regulation of lipid homeostasis in the liver. Through physiological, molecular, biochemical, and genetic rescue studies, we have identified a key role of Dyrk1b in promoting DNL by triggering the activation of mTORC2, the central regulator of hepatic lipogenesis (17).

## Results

### Dyrk1b is upregulated in the liver of mice fed a high-calorie diet and in human patients with NASH.

**Figure 1:**
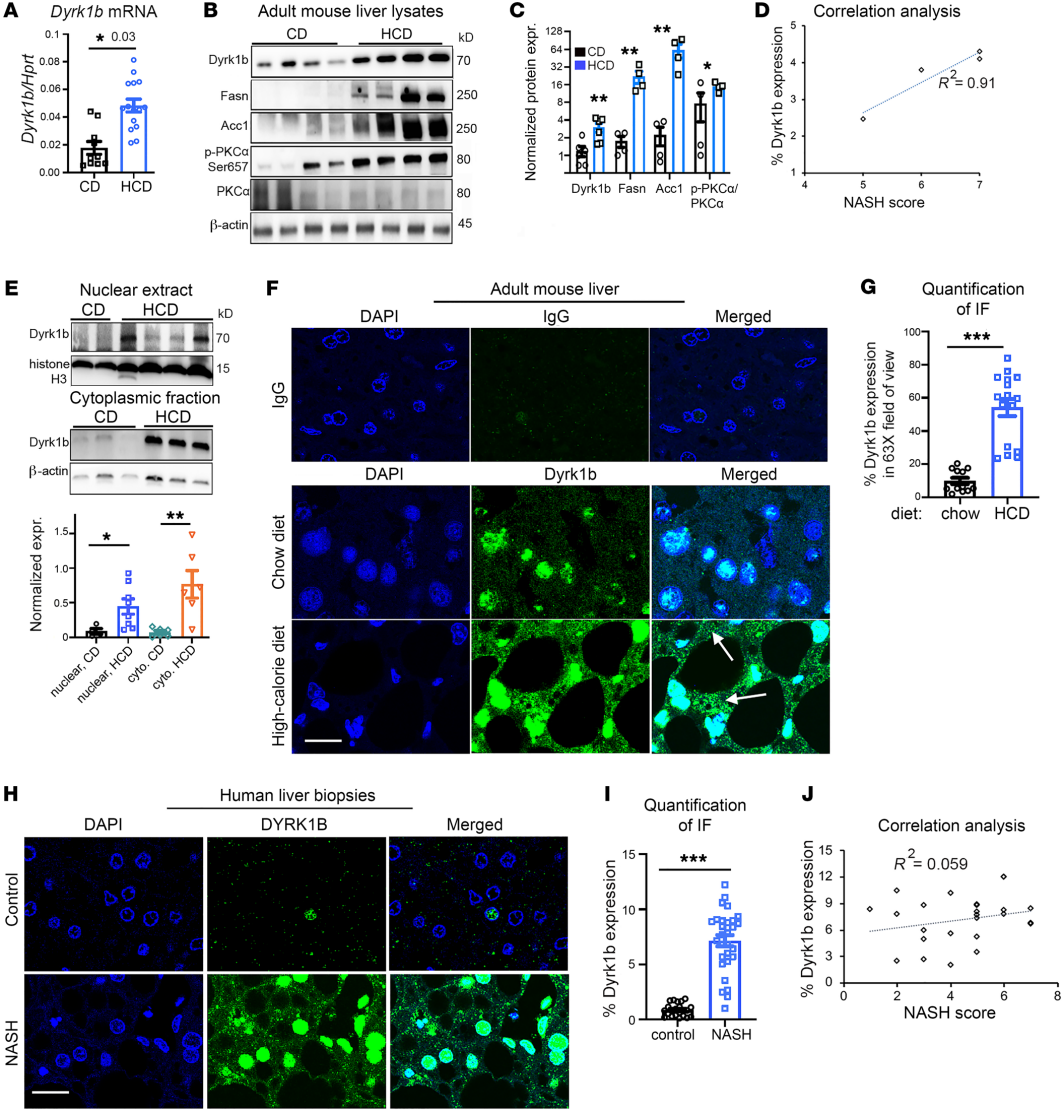
Dyrk1b is upregulated in the liver of mice with diet-induced fatty liver disease and in human NASH. (**A**) *Dyrk1b* transcripts normalized to *Hprt* in the indicated conditions. Henceforth, each dot in the dot plots represents biological replicates; *n >* 8 mice each, unpaired *t* test, 2-tailed. (**B** and **C**) Western blot (WB) analysis and quantification of specified liver proteins in mice fed CD or HCD. Henceforth, each lane of WBs represents biological replicates; *n =* 4 mice each, unpaired *t* test, 2-tailed. (**D**) Correlation analysis between NASH score and Dyrk1b expression in the liver of HCD-fed mice. (**E**) WB and quantification of Dyrk1b levels in the nuclear (top) and cytoplasmic extracts (bottom) in the liver of mice fed with CD or HCD; *n >* 6 each, unpaired *t* test, 2-tailed. (**F** and **G**) Dyrk1b protein expression visualized by immunofluorescence in the liver of CD- or HCD-fed mice. IgG was used as control (top row); *n =* 5 mice each group. Arrows indicate cytoplasmic expression. Unpaired *t* test, 2-tailed. (**H** and **I**) Representative images and quantification of DYRK1B expression in the liver biopsies of patients with NASH versus controls; *n =* 20 controls, *n =* 27 NASH samples, unpaired *t* test, 2-tailed, Welch-corrected. (**J**) Correlation analysis between NASH score and percentage Dyrk1b expression in the human NASH samples (*n =* 22) from **I**. Scale bars: 20 μm. *P ≤ 0.05, **P ≤ 0.01, ***P ≤ 0.001.

Subsequent to the identification of strong linkage between gain-of-function mutations in *DYRK1B* and MetS (24), we sought to determine the global role of the encoded protein in disease pathogenesis. We reasoned that the major traits of the mutation carriers, hypertriglyceridemia, fatty liver disease, and T2D, are the consequence of altered hepatic glucose and lipid metabolism, and embarked on characterizing the hepatic function of Dyrk1b. We fed mice a diet consisting of equivalent calories from fats and sucrose (40% each), hereafter referred to as high-calorie diet (HCD), to stimulate hepatic triacylglycerol (TAG) accumulation via increased DNL by dietary carbohydrates, and increased uptake of dietary fat (1). C57BL/6J mice on 3-month HCD exhibited increased fat accumulation in the liver compared with mice on chow diet (CD) (Supplemental Figure 1A; supplemental material available online with this article; https://doi.org/10.1172/JCI153724DS1). Strikingly, *Dyrk1b* transcript (Figure 1A) and protein levels (Figure 1, B and C) were elevated in the liver of HCD- versus CD-fed mice and highly correlated with NASH severity (Figure 1D). The hepatic Dyrk1b levels were also increased in mice fed with diet consisting of 60% calories from fat (Supplemental Figure 1B). The Dyrk1b expression was lost when antibody was preblocked with the corresponding peptide, confirming specificity of the antibody (Supplemental Figure 1C and Supplemental Figure 5H). Accordingly, the levels of the DNL enzymes Fasn and Acc1 and p-PKCα/PKCα, a readout for mTORC2 activation (17), were increased in the liver of HCD- versus CD-fed mice (Figure 1, B and C) and positively correlated with degree of NASH severity (Supplemental Figure 1, D–F). Dyrk1b was increased in the HCD-fed mice in both nuclear (Figure 1, E–G, and Supplemental Figure 1G) and cytoplasmic compartments (Figure 1, E–G, and Supplemental Figure 1G) as determined by cellular fractionation and immunofluorescence imaging. These data are consistent with the previously reported bipartite expression of Dyrk1b in C2C12 myoblasts (25, 29). HCD increased Dyrk1b levels in the hepatocytes (Supplemental Figure 1, H–J), and in the macrophages (Supplemental Figure 1, K–M). The vascular smooth muscle cells (VSMCs) stained positive for Dyrk1b in both CD- and HCD-fed mouse livers, although the overall levels were higher in the latter (Supplemental Figure 1, N–P). Taken together, these findings indicate that a diet enriched with fat and/or carbohydrates can upregulate Dyrk1b expression throughout the liver.

We next examined the relevance of DYRK1B to human disease by exploring its protein levels in patients diagnosed with NASH. We obtained deidentified liver samples from patients with biopsy-proven NASH and compared them with liver samples of individuals without NASH or diabetes (Supplemental Table 1). Using the same antibody as described above, an average of 7.0-fold increase in the DYRK1B levels was observed in the liver biopsies of NASH patients versus controls by immunofluorescence (Figure 1, H and I). The elevated levels of liver Dyrk1b strongly correlated with NASH diagnosis but not with its severity, implicating its role in disease pathogenesis in humans as opposed to disease progression (Figure 1J). The expression of the DNL enzyme Acc1 was also increased in the NASH specimens (Supplemental Figure 1Q). Altogether, Dyrk1b levels are increased in diet-induced fatty liver disease in mice and in humans with NASH. These findings prompted further studies to examine the causal role of Dyrk1b in disease pathogenesis of NAFLD.

### Elevated hepatic Dyrk1b levels cause hyperlipidemia and hepatic steatosis in a kinase-independent fashion.

**Figure 2:**
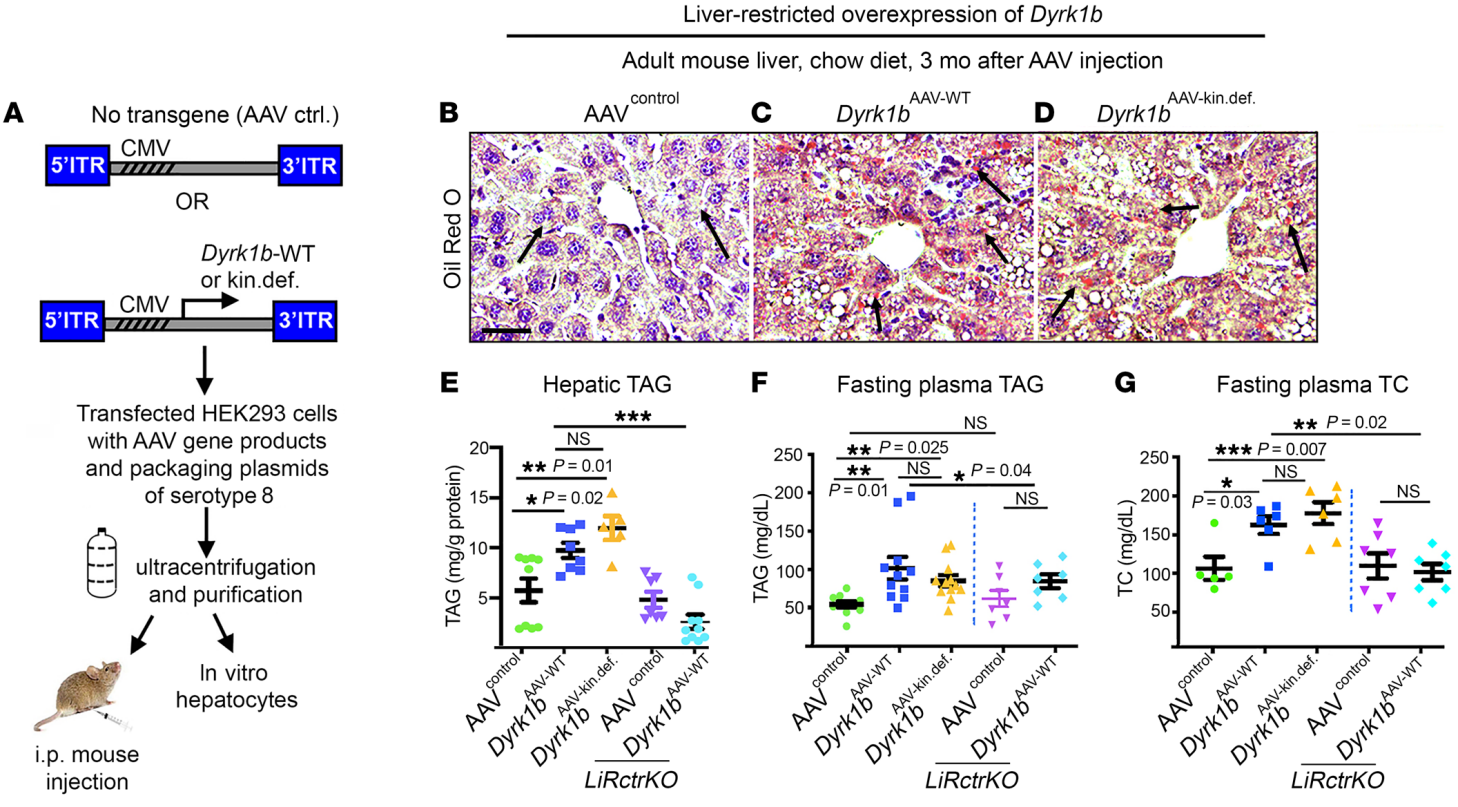
Dyrk1b regulates hepatic TAG in a kinase-independent manner. (**A**) Schematic representation of generation of AAV8 for increasing Dyrk1b levels in the mouse liver. (**B**–**E**) Oil Red O (ORO) staining, counterstained with hematoxylin (**B**–**D**) and total hepatic TAG (**E**) in the designated mice on CD after 6-hour fast; *n >* 6 each, 1-way ANOVA, Tukey’s post hoc test. Arrows in **B**–**D** indicate neutral lipid staining. (**F**) Fasting plasma TAG of the indicated mice on CD after 6-hour fast; *n >* 6 mice each, 1-way ANOVA, Tukey’s post hoc test. (**G**) Fasting TC on CD after 6-hour fast; *n >* 5 mice each, 1-way ANOVA, Tukey’s post hoc test.*P ≤ 0.05, **P ≤ 0.01, ***P ≤ 0.001. Scale bar: 150 μm.

To determine the causal role of Dyrk1b in NAFLD, we overexpressed Dyrk1b in the liver of mice by adeno-associated virus serotype 8 (AAV8). AAV8 is touted for efficient, specific, and long-term liver-targeted gene therapy in both rodents and humans (30, 31). The AAV8 containing empty vector (AAV^control^) or wild-type (*Dyrk1b^AAV-WT^*) or kinase-defective Dyrk1b cDNA (*Dyrk1b^AAV-kin.def^*) was transduced in vivo (Figure 2A). The kinase-defective *Dyrk1b^K140R,Y273F^* (24, 32) curtailed the phosphorylation of recombinant human Fkhr (rhFkhr) at Ser329, a previously established kinase-dependent modification by Dyrk1b (ref. 33 and Supplemental Figure 2A). The AAV8 was titered to increase *Dyrk1b* mRNA levels 2-fold (Supplemental Figure 2, B–D), which is less than 50% of the increase we observed in the liver of HCD- versus CD-fed mice (compare with Figure 1G, 5-fold change HCD vs. CD). As expected, other organs involved in maintaining lipid and glucose metabolism, such as pancreas, adipose tissue, or skeletal muscle, showed no change in the expression of *Dyrk1b* or *Dyrk1a*, a human paralog of *Dyrk1b* (Supplemental Figure 2B), in *Dyrk1b^AAV-WT^* versus AAV^control^ mice. Next, to trace the tropism of the virus in the different cell types in the liver, we transduced GFP using AAV8. The albumin-positive hepatocytes and VSMCs showed GFP transduction while F4/80-positive Kupffer cells and CD31-positive endothelial cells showed minimal transduction with AAV8 (Supplemental Figure 3, A–D). Altogether, we obtained relatively specific transduction of Dyrk1b in the hepatocytes in the liver.

Remarkably, both *Dyrk1b^AAV-WT^* and *Dyrk1b^AAV-kin.def^* mouse liver demonstrated 1.82-fold and 2.3-fold increase in hepatic TAG content, respectively, as compared with the AAV^control^ mice simply on a CD (Figure 2, B–E). Accordingly, the fasting plasma TAG was increased 1.85-fold in *Dyrk1b^AAV-WT^* and 1.54-fold in *Dyrk1b^AAV-kin.def^* mice versus AAV^control^ mice (Figure 2F). The fasting plasma total cholesterol (TC) was elevated 1.34-fold in *Dyrk1b^AAV-WT^* and 1.47-fold in *Dyrk1b^AAV-kin.def^* mice versus AAV^control^ (Figure 2G). A prolonged HCD for 9 months after AAV8 injection caused a significant increase in hepatic TAG and liver weight in *Dyrk1b^AAV-WT^* mice (Supplemental Figure 2, E–H). The total body weight was slightly reduced, while percentage liver weight, percentage lean mass, percentage fat mass, and food and water intake remained unchanged (Supplemental Figure 2, I–N) in *Dyrk1b^AAV-WT^* versus AAV^control^ mice, fed with CD for 3 months. The relative adipose tissue weight (Supplemental Figure 2O) and the plasma nonesterified free fatty acid (NEFA) levels (Supplemental Figure 2P) were similar between *Dyrk1b^AAV-WT^* and AAV^control^, diminishing the likelihood of a contribution of adipose tissue to the hepatic TAG in *Dyrk1b^AAV-WT^* liver. Altogether, these data indicate that Dyrk1b causes steatosis and hyperlipidemia in mice independent of its kinase activity.

To verify that the observed effects of Dyrk1b are specific to the hepatocytes, we overexpressed *Dyrk1b* in primary hepatocytes of WT and *Dyrk1b^–/–^* mice. The transduction of both *Dyrk1b^AAV-WT^* and *Dyrk1b^AAV-kin.def^* increased the total TAG levels in *Dyrk1b^–/–^* and WT hepatocytes (Supplemental Figure 4, A–D). Altogether, these data indicate that Dyrk1b causes steatosis directly in the hepatocytes irrespective of its kinase activity.

### Reduced Dyrk1b protects against diet-induced hepatic steatosis and hypertriglyceridemia.

**Figure 3:**
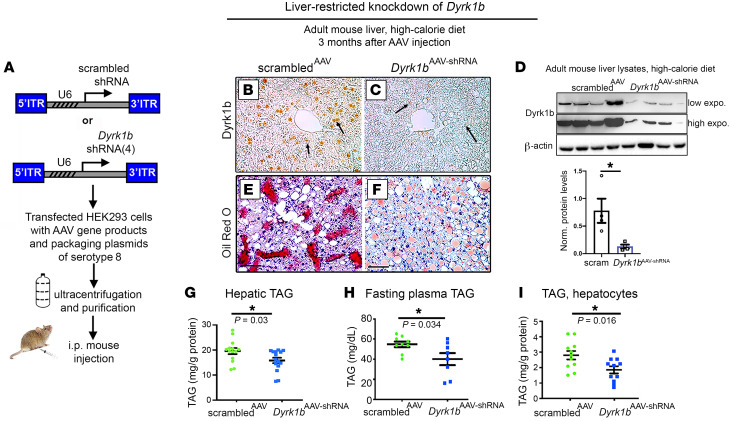
Knockdown of *Dyrk1b* protects against hepatic steatosis and hyperlipidemia. (**A**) Schematic representation of knockdown of *Dyrk1b* using shRNAs targeted to the liver by AAV8. (**B**–**D**) *Dyrk1b* expression in *Dyrk1b^AAV-shRNA^* versus scrambled^AAV^ visualized by HRP-mediated IHC in the liver (**B** and **C**) and WB (**D**); *n >* 5 mice each for **B** and **C**. The mice were fed HCD for 3 months. Unpaired *t* test, 2-tailed. (**E**–**G**) ORO staining, counterstained with hematoxylin, and total hepatic TAG (**G**) in the liver of indicated mice, fed HCD for 3 months and fasted for 6 hours; *n >* 10 each, unpaired *t* test, 2-tailed. (**H**) Plasma TAG after 6-hour fast in *Dyrk1b^AAV-shRNA^* versus scrambled^AAV^ mice; *n >* 7 each, unpaired *t* test, 2-tailed. (**I**) Primary hepatocytes transduced by AAV8 containing either scrambled or *Dyrk1b* shRNA at an MOI of 60; *n =* 11 per group, unpaired *t* test, 2-tailed. Scale bars: 150 μm. *P ≤ 0.05.

Next, we determined whether knocking down hepatic *Dyrk1b* would confer protection against NAFLD. We used 4 different shRNAs against *Dyrk1b* mRNA (*Dyrk1b^AAV-shRNA^*) that were each cloned into AAV8 vectors, packaged into viral particles, purified, and coinjected i.p. into each mouse (Figure 3A). The target sequences of the shRNAs were 100% specific for mouse *Dyrk1b* and were spread through different exons of the mRNA (Supplemental Table 2). The *Dyrk1b^AAV-shRNA^* reduced hepatic Dyrk1b levels in comparison with the mice transduced with scrambled shRNA (scrambled^AAV^; Figure 3, B–D, and Supplemental Figure 5A). As before, the expression of *Dyrk1b* in skeletal muscle, adipose tissue, and pancreas and hepatic *Dyrk1a* levels remained unchanged in *Dyrk1b^AAV-shRNA^* mice (Supplemental Figure 5, B and C). The expression levels of potential off-targets of *Dyrk1b* shRNAs, as predicted by percentage DNA homology to the shRNA sequence (Supplemental Table 2), displayed no changes in *Dyrk1b^AAV-shRNA^* mouse liver (Supplemental Figure 5A).

On HCD, *Dyrk1b^AAV-shRNA^* mice were substantially protected from hepatic steatosis (Figure 3, E–G) and fasting hypertriglyceridemia versus scrambled^AAV^ mice (Figure 3H). The isolated hepatocytes from WT mice, transduced with *Dyrk1b^AAV-shRNA^*, showed significantly lower TAG content compared with scrambled^AAV^ (Figure 3I), indicating a hepatocyte-specific cell-autonomous effect. No changes were noted in TC (Supplemental Figure 5D), the relative liver weight (Supplemental Figure 5E), or the fasting plasma NEFA (Supplemental Figure 5F) in the *Dyrk1b^AAV-shRNA^* mice. In conclusion, *Dyrk1b* knockdown confers significant protection against diet-induced hepatic steatosis and hypertriglyceridemia.

Next, we examined the effect of global *Dyrk1b* disruption on the development of fatty liver. A gene trap cassette was introduced consisting of a splice acceptor site followed by a polyadenylation site in intron 2 of the endogenous *Dyrk1b* locus (Supplemental Figure 5G). *Dyrk1b^–/–^* mice were born in Mendelian ratios and appeared healthy. The cryptic splice acceptor site was substantially effective in generating knockouts, causing 90% reduction in liver Dyrk1b protein levels (Supplemental Figure 5, H and I). The hepatic TAG content was significantly reduced in *Dyrk1b^–/–^* liver on CD at 3 months (Supplemental Figure 5, J and K) and 5 months (Supplemental Figure 6A). However, *Dyrk1b^–/–^* mice on HCD were not protected against hyperlipidemia or hepatic steatosis (Supplemental Figure 7, A and B), in contrast to *Dyrk1b^AAV-shRNA^* mice (Figure 3, E–G). We reasoned that these differences were due to extrahepatic effects of *Dyrk1b*, since *Dyrk1b^–/–^* is a global knockout. *Dyrk1b^–/–^* mice on HCD, as opposed to *Dyrk1b^AAV-shRNA^*, had lower Dyrk1b expression in adipose tissue (Supplemental Figure 7, C vs. D) and showed higher adipose tissue weight (Supplemental Figure 7, F vs. E and G) and higher fasting NEFA (Supplemental Figure 7H vs. Supplemental Figure 5F) compared with corresponding littermate controls. To demonstrate the autonomous role of Dyrk1b in hepatic lipogenesis, we examined TAG accumulation in isolated *Dyrk1b^–/–^* hepatocytes from CD-fed mice and administered 2.5 mM glucose in full media (see Methods) for 5 days to stimulate lipogenesis. As predicted, *Dyrk1b^–/–^* hepatocytes showed significantly lower TAG content compared with WT hepatocytes (Supplemental Figure 5, L and M). No changes were observed in total adipocyte area in *Dyrk1b^–/–^* or *Dyrk1b^AAV-shRNA^* mice (Supplemental Figure 7, I–K). The hepatic TAG secretion (Supplemental Figure 5N), body weight, percentage lean mass, TC, and liver weight were not different (Supplemental Figure 5, O–R) while percentage fat mass and food and water consumption were modestly increased (Supplemental Figure 5, S–U; *n >* 7 for each genotype) in *Dyrk1b^–/–^* mice versus littermate controls. Altogether, these data indicate that hepatic, but not global, reduction of *Dyrk1b* confers significant protection against diet-induced hepatic steatosis.

### Dyrk1b stimulates hepatic DNL, TAG secretion, and fatty acid uptake.

**Figure 4:**
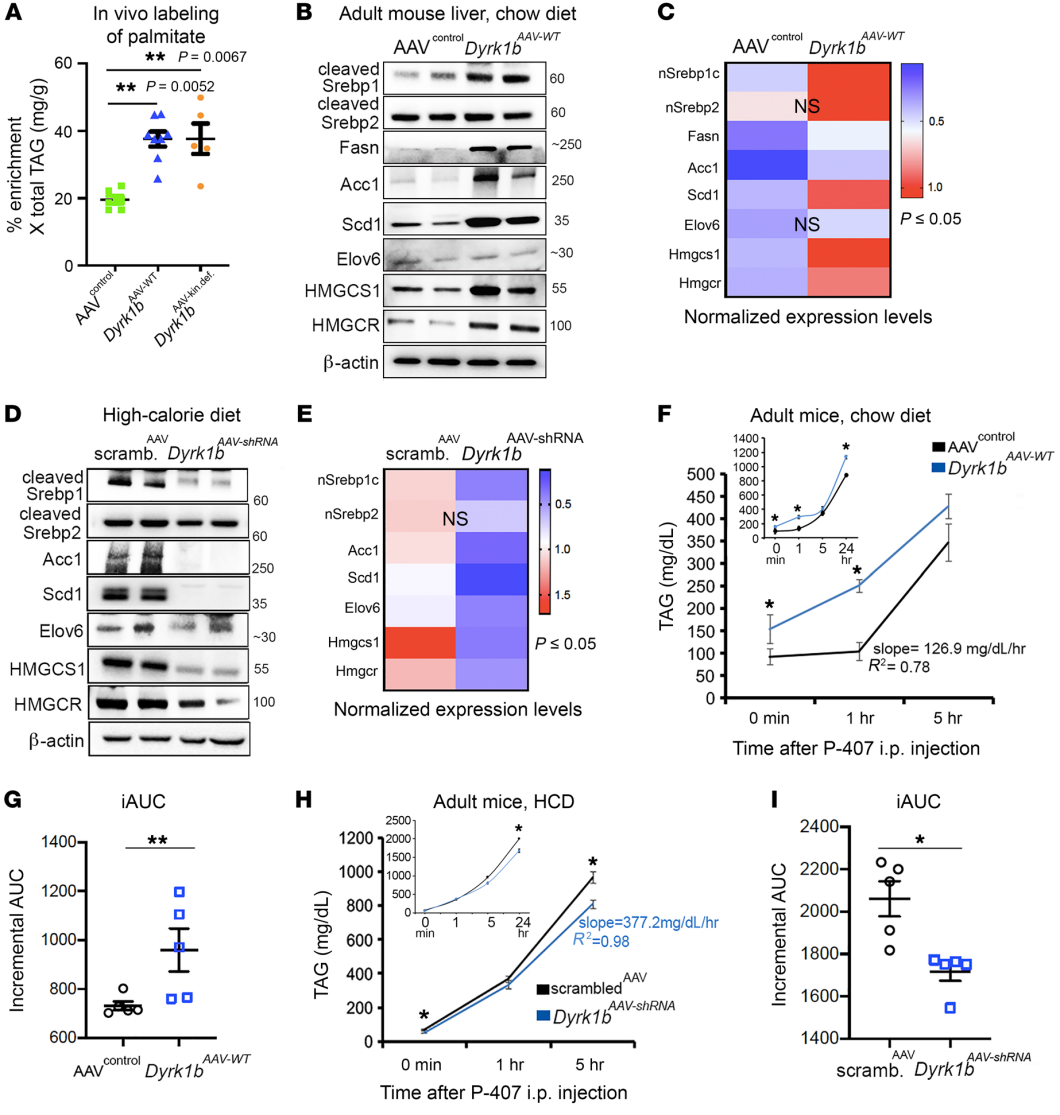
Dyrk1b promotes DNL in the liver. (**A**) Graph showing the absolute amount of deuterium-labeled (M+1 and M+2) palmitate to unlabeled palmitate quantified by gas chromatography–mass spectrometry in the indicated genotypes. The contribution of naturally occurring M+1 and M+2 palmitates was subtracted. Percent enrichment × TAG (mg/g): AAV^control^, 19.55 ± 1.18, *n =* 9; *Dyrk1b^AAV-WT^*, 37.55 ± 2.75, *n =* 8; *Dyrk1b^AAV-kin.def^*, 38.19 ± 5.79, *n =* 5; 1-way ANOVA, Tukey’s post hoc test. (**B** and **C**) Expression and quantification of designated proteins in the liver of the indicated mice fed with CD, after 6-hour fast; *n =* 4 mice each genotype, unpaired *t* test, 2-sided. (**D** and **E**) Expression and quantification of designated proteins in the liver of the indicated mice fed with HCD after 6-hour fast; *n =* 4 mice each genotype, unpaired *t* test, 2-sided. (**F** and **G**) Rate of hepatic triglyceride secretion (**F**) and incremental area under the curve (iAUC) (**G**) upon i.p. administration of weight-normalized poloxamer-407 (P-407) in the designated mice on CD for 3 months. iAUC values were calculated by subtraction of baseline (0 minutes) values from other time points, to minimize the bias from high plasma TAG at baseline in *Dyrk1b^AAV-WT^* mice. The mice were fasted for 6 hours and plasma collected at the indicated times after P-407 injection. AAV^control^, 126.9 mg/dL/h; *Dyrk1b^AAV-WT^*, 136.9 mg/dL/h; *n* > 5 each group, unpaired *t* test, 2-sided. (**H** and **I**) Rate of hepatic triglyceride secretion (**H**) and iAUC (**I**) upon administration of P-407 in the designated mice on HCD for 3 months; scrambled^AAV^, 448.1 mg/dL/h; *Dyrk1b^AAV-shRNA^*, 377.2 mg/dL/h; *n =* 5 each genotype, unpaired *t* test, 2-sided. *P ≤ 0.05, **P ≤ 0.01.

Since patients with T2D and NASH exhibit increased DNL (7, 34), we investigated whether Dyrk1b-induced steatosis is due to increased hepatic DNL. The de novo synthesized lipids were pulse-labeled by feeding of mice with 5% deuterium oxide in the drinking water for a total of 7 days along with a 70% fructose diet to stimulate DNL as previously described (35). The deuterium-labeled palmitate was quantified by gas chromatography–mass spectrometry (GC-MS). The absolute amount of palmitate synthesized by DNL and percentage enrichment of deuterated palmitate in the liver were significantly higher in both *Dyrk1b^AAV-WT^* and *Dyrk1b^AAV-kin.def^* versus AAV^control^ mice (Figure 4A and Supplemental Figure 2Q). Consistent with increased DNL, the expression levels of enzymes in the DNL pathway such as cleaved Srebp1 and its downstream targets Fasn, Acc1, and Scd1 (except for Elov6), and enzymes in the cholesterol biosynthesis pathway such as HMGCR and HMGCS1, were significantly higher in *Dyrk1b^AAV-WT^* (Figure 4, B and C, and additional biological replicates in Supplemental Figure 8A) and *Dyrk1b^AAV-kin.def^* versus AAV^control^ mice on CD (Supplemental Figure 8C, rows 1–5). Conversely, cleaved Srebp1, Acc1, Scd1, HMGCS1, and HMGCR were significantly reduced in the liver of *Dyrk1b^AAV-shRNA^* mice versus scrambled^AAV^ on HCD (Figure 4, D and E, and additional biological replicates in Supplemental Figure 8B). Overall, these experiments indicate that Dyrk1b promotes hepatic DNL and stimulates the expression of rate-limiting enzymes in the lipogenesis pathway.

We subsequently measured hepatic TAG secretion after inhibiting LPL activity by i.p. injection of poloxamer-407. The rate of TAG secretion was significantly higher in *Dyrk1b^AAV-WT^* versus AAV^control^ mice on CD (Figure 4, F and G), and significantly reduced in *Dyrk1b^AAV-shRNA^* mice versus scrambled^AAV^ controls on HCD (Figure 4, H and I). No changes were detected in microsomal triglyceride transfer protein (MTTP) and apolipoprotein B (ApoB) in *Dyrk1b^–/–^* mouse liver (Supplemental Figure 9, A and B). MTTP was considerably increased while there was a subtle reduction of ApoB levels in *Dyrk1b^AAV-WT^* mouse liver in comparison with WT mice (Supplemental Figure 9, C and D). Taken together, these data indicate that Dyrk1b stimulates hepatic DNL, which in turn increases TAG secretion, leading to steatosis and hyperlipidemia.

**Figure 5:**
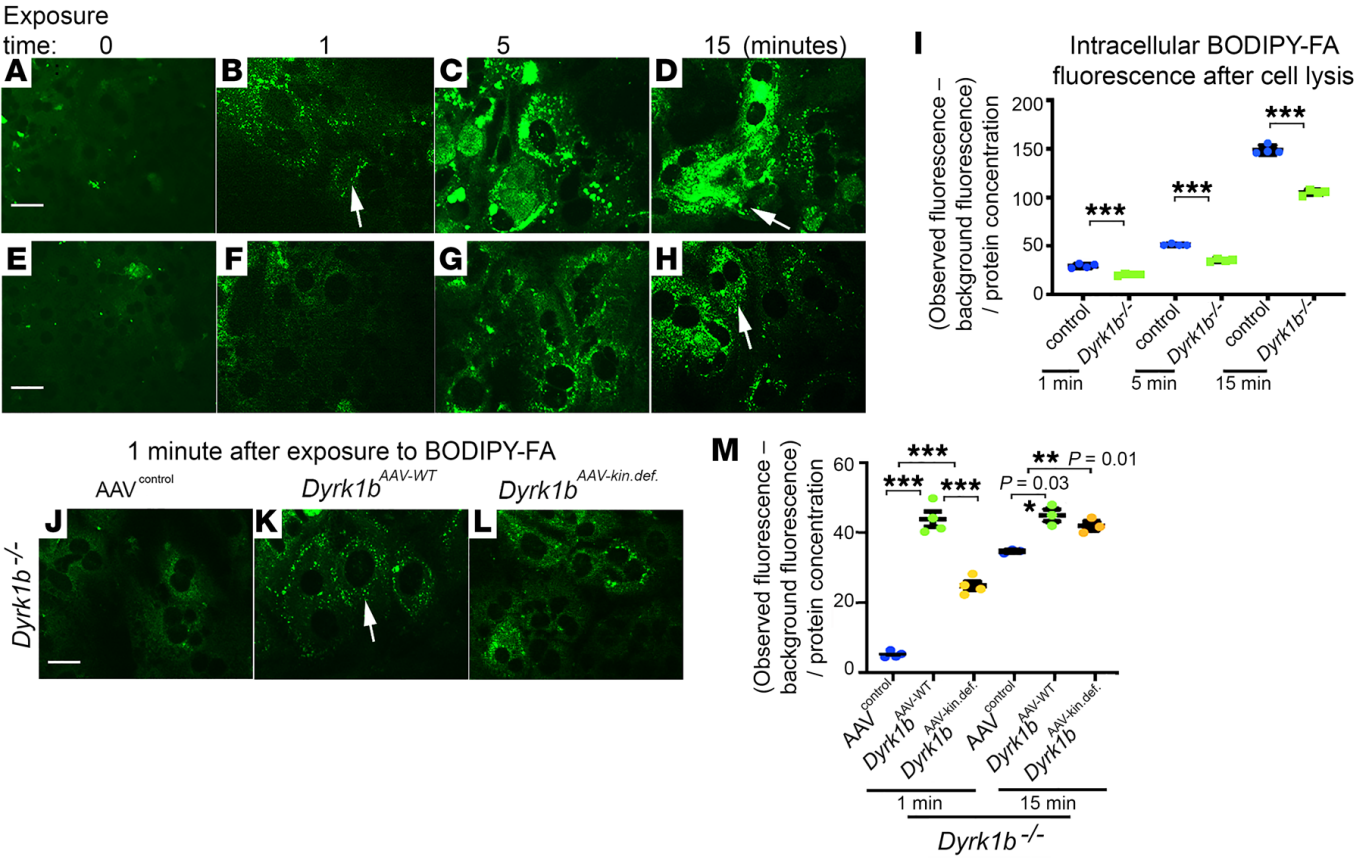
Dyrk1b increases FA uptake in the hepatocytes. (**A**–**H**) Representative confocal images from littermate controls and *Dyrk1b^–/–^* hepatocytes at indicated time points after addition of BODIPY-FA; *n =* 5 mice each genotype, *n =* 4 technical replicates. (**I**) Quantification of intracellular fluorescence in controls and *Dyrk1b^–/–^* hepatocytes measured by the microplate reader after cell lysis at the indicated time points. The background fluorescence (prior to addition of BODIPY-FA) was subtracted and normalized to the total protein content. Unpaired *t* test, 2-sided, *n =* 5 mice each genotype, *n =* 4 technical replicates. (**J**–**L**) Representative confocal images showing BODIPY-FA uptake 1 minute after addition in *Dyrk1b^–/–^* hepatocytes transduced with AAV8 containing empty vector (AAV^control^) or *Dyrk1b^WT^* or *Dyrk1b^kin.def^* virus, at an MOI of 60. The FA uptake experiment was performed 72 hours after virus transduction, to allow for sufficient transcription. *n =* 2 mice each condition, *n =* 4 technical replicates. (**M**) Quantification of intracellular fluorescence, in the indicated genotypes; 1-way ANOVA, Tukey’s post hoc test. White arrows indicate the intracellular fluorescence detected by BODIPY-FA. Scale bars: 150 μm. **P ≤ 0.01, ***P ≤ 0.001.

We then examined the potential effect of Dyrk1b on fatty acid (FA) uptake. We visualized the uptake of fluorescent BODIPY-conjugated C16 FA by hepatocytes and quantified the intracellular fluorescence after cell lysis. We examined FA uptake starting 1 minute after addition of BODIPY-C16 to curtail any effects arising from the incorporation of BODIPY-C16 into neutral lipid pool. The uptake of BODIPY-FA increased over a period of 15 minutes in the hepatocytes from littermate control mice (Figure 5, A–D) but was significantly reduced in *Dyrk1b^–/–^* hepatocytes at 1, 5, and 15 minutes after addition of BODIPY-FA (Figure 5, E–I). The *Dyrk1b^–/–^* hepatocytes transduced with *Dyrk1b^AAV-WT^* showed higher FA uptake within 1 minute of addition of BODIPY-FA (Figure 5, J and K) compared with AAV^control^. The *Dyrk1b^–/–^* hepatocytes transduced with *Dyrk1b^AAV-kin.def^* virus also showed higher FA uptake at 1 minute after addition of BODIPY-FA compared with AAV^control^, but lower compared with *Dyrk1b^AAV-WT^*, suggesting that FA uptake is partially dependent on kinase activity of Dyrk1b (Figure 5, J–M). Similarly, a time course of FA uptake in the WT hepatocytes transduced with *Dyrk1b^AAV-WT^* virus displayed increased FA uptake compared with AAV^control^ in a partially kinase-dependent manner (Supplemental Figure 10, A–J). A Seahorse assay revealed that endogenous basal FA oxidation was significantly decreased in *Dyrk1b^–/–^* hepatocytes versus littermate controls (Supplemental Figure 11, A and B) and was significantly increased by *Dyrk1b* in a kinase-dependent manner (Supplemental Figure 11, C and D). This finding indicated a direct correlation between the rate of FA uptake and FA oxidation and that Dyrk1b-induced steatosis is caused by increased DNL and FA uptake and not reduced FA oxidation.

### Rictor, the obligate mTORC2 subunit, is the upstream regulator of proteome altered by Dyrk1b.

**Figure 6:**
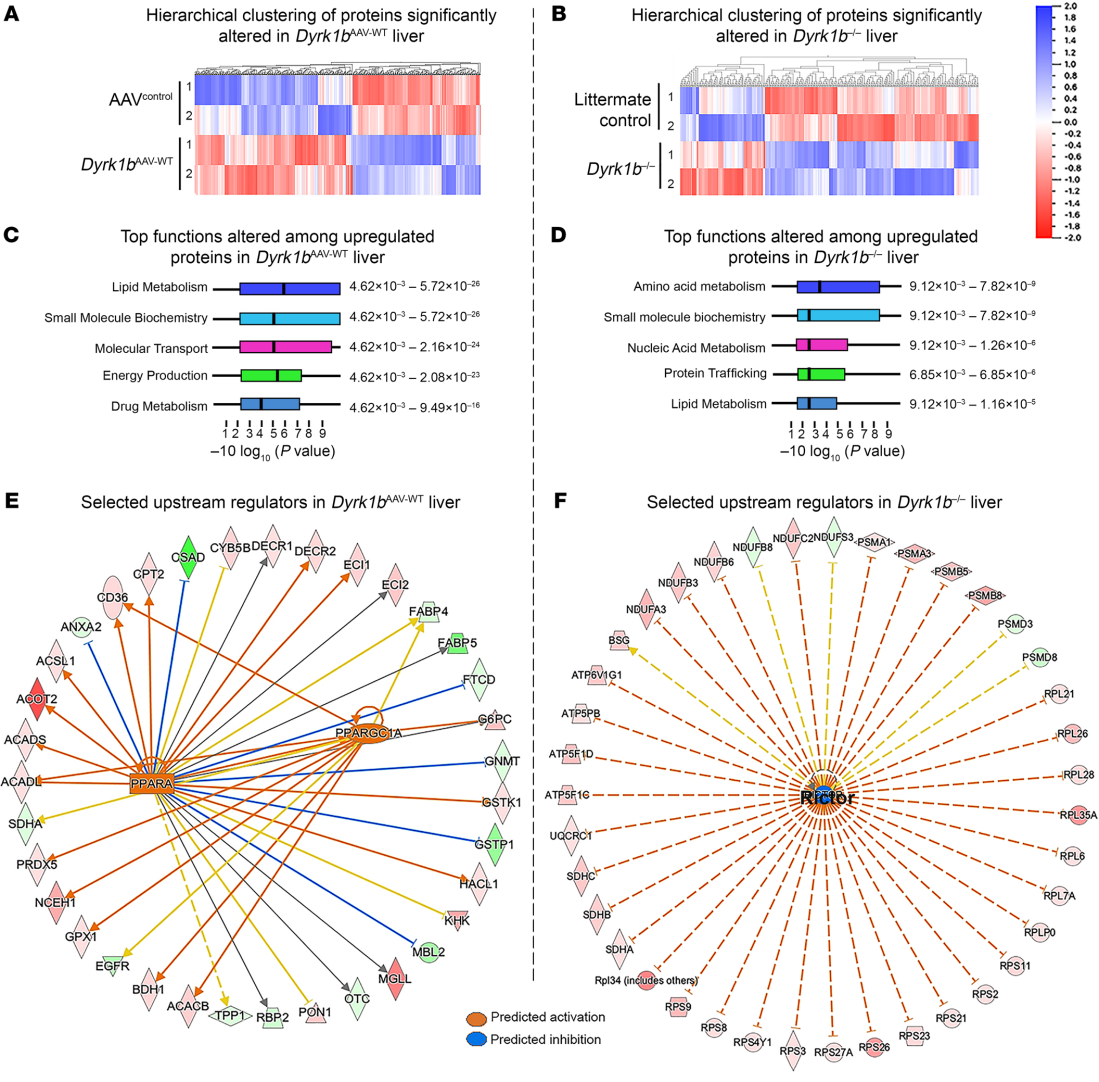
Analysis of globally altered proteomes in the liver upon changes in *Dyrk1b* levels. (**A** and **B**) Representative heatmaps displaying hierarchical clustering of significantly altered proteome in the liver of designated mice; *n =* 2 are displayed for each condition; *n =* 3 biological replicates were analyzed for each genotype. The fold changes relative to respective controls were calculated, and the significantly altered proteins were determined using the following thresholds: *P* < 0.05, unpaired *t* test, FDR < 0.05, Benjamini-Hochberg corrected. The heatmaps were generated by Qlucore Omics software. The tissues were processed by liquid chromatography–tandem mass spectrometry. (**C** and **D**) The altered functions predicted by IPA for *Dyrk1b^AAV-WT^* (**C**) and *Dyrk1b^–/–^* (**D**). (**E** and **F**) Top selected upstream regulators of proteins that are altered in *Dyrk1b^AAV-WT^* (**E**) and *Dyrk1b^–/–^* (**F**) liver predicted by IPA.

Next, we performed expression proteomics in CD-fed *Dyrk1b^AAV-WT^* and *Dyrk1b^–/–^* liver to identify Dyrk1b-dependent global alterations in the signaling pathways causing NAFLD (Figure 6, A and B). The *Dyrk1b^–/–^* mice on CD showed significant reduction in hepatic TAG (Supplemental Figure 5, J and K) and were protected against Dyrk1b-dependent pathways that induce NAFLD. Separate analyses were performed for proteins that are upregulated and those that are downregulated in *Dyrk1b^AAV-WT^* and *Dyrk1b^–/–^* liver. Ingenuity Pathway Analysis (IPA) identified proteins involved in lipid metabolism as being altered in the liver of both *Dyrk1b^AAV-WT^* and *Dyrk1b^–/–^* mice (Figure 6, C and D, and Supplemental Tables 3 and 4). Within the category of lipid metabolism, lipid synthesis (*z* score = 3.51), uptake (*z* score = 2.36), and β-oxidation of FA (*z* score = 2.617) were increased in *Dyrk1b^AAV-WT^* versus AAV^control^ (Supplemental Table 5). Conversely, in *Dyrk1b^–/–^* liver, lipid synthesis and accumulation showed negative *z* scores of –1.6 and –0.647, respectively (Supplemental Table 6). These patterns are consistent with the experimental observations that Dyrk1b stimulates lipogenesis and FA uptake along with a compensatory increase in oxidation of FAs. The downregulated pathways in *Dyrk1b^AAV-WT^* liver and upregulated pathways in *Dyrk1b^–/–^* liver are interesting on their own but are beyond the scope of this article (Supplemental Tables 3 and 4).

Next, IPA was used to predict the potential upstream regulators of the altered proteins in the *Dyrk1b^AAV-WT^* and *Dyrk1b^–/–^* liver. All the significantly altered proteins were used as input to predict potential upstream regulators. Peroxisome proliferator–activated receptor-γ coactivator 1α (PPARGC1a) (*P* = 4.8 × 10^–4^, *z* score = 2.27) and PPARα (PPARA) (*P* = 1.72 × 10^–11^, *z* score = 2.22), two sentinel regulators of hepatic lipid metabolism, were among the top upstream regulators of *Dyrk1b^AAV-WT^* liver proteome (Figure 6E and Supplemental Table 7). PPARGC1a activation was previously associated with upregulation of lipogenesis (36) and VLDL secretion (37), and PPARA activation is associated with an adaptive increase in β-oxidation in high-fat diet–fed mice (38). In the *Dyrk1b^–/–^* liver, Rictor, an obligate mTORC2 subunit (19), was the most significantly inhibited upstream regulator (*P* = 6.74 × 10^–25^, *z* score = –4.54; Figure 6F and Supplemental Table 8). Given the central role of mTORC2 in promoting hepatic lipogenesis (17–19), we focused on its regulation by Dyrk1b as follows below. In addition to Rictor, the proteins regulated by insulin receptor (*P* = 6.1 × 10^–6^, *z* score = 3.13) were predicted to increase in *Dyrk1b^–/–^* mice (Supplemental Table 8).

### Dyrk1b stimulates phosphorylation and activation of mTORC2 in a kinase-independent manner.

**Figure 7:**
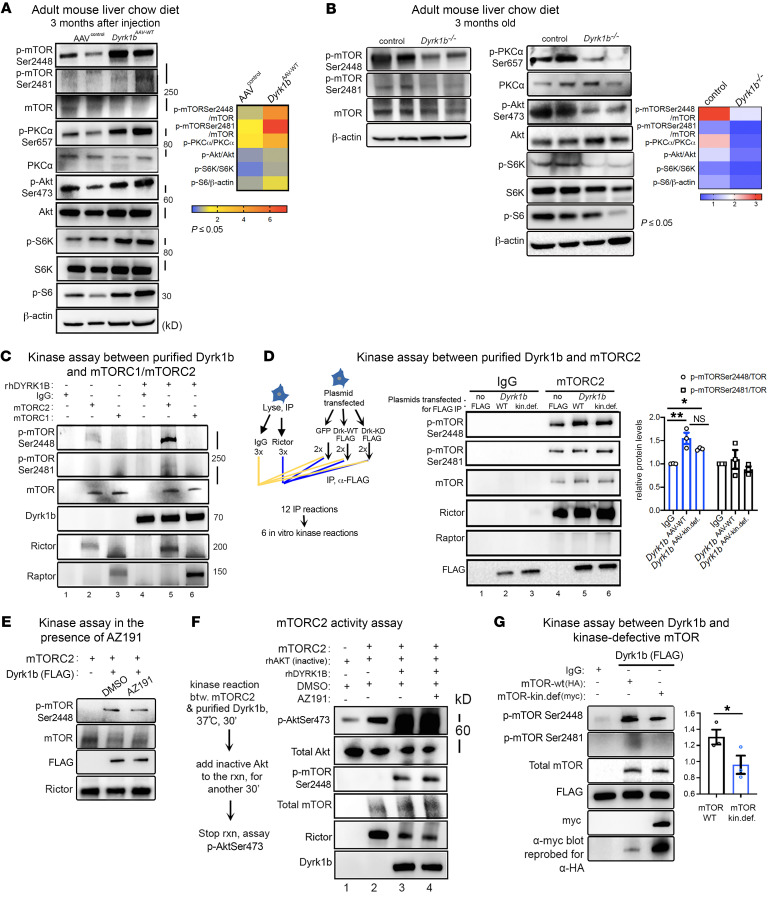
Dyrk1b activates mTORC2 in a kinase-independent manner. (**A** and **B**) Expression and quantification of proteins by WB indicating mTORC1 and mTORC2 activity in the liver of adult *Dyrk1b^AAV-WT^* and *Dyrk1b^–/–^* mice and their corresponding littermate controls, fed with CD and after 6-hour fast; unpaired *t* test, 2-tailed. (**C**) WB displaying kinase assay between rhDYRK1B and mTOR complexes; *n =* 3 experiments. (**D**) Schematic (left) displaying the experimental design and WB (right) showing the kinase assay between IgG/Dyrk1b (WT or kin.def) and mTORC2; *n =* 3 experiments, 1-way ANOVA, Tukey’s post hoc test. (**E**) Kinase assay between immunoprecipitated Dyrk1b and mTORC2 in the presence of either DMSO or AZ191; *n =* 3 experiments. (**F**) rhDYRK1B-mediated activation of mTORC2 assayed by p-AktSer473. The kinase assay was carried out between mTORC2 and rhDYRK1B at 37°C for 30 minutes as before, followed by addition of purified, recombinant inactive Akt (rhAkt) for another 30 minutes. DMSO or AZ191 (10 μm) was added as before. *n =* 4 experiments. See Supplemental Figure 13B (first row) for more replicates. (**G**) WB showing results of kinase reaction between Dyrk1b and IgG/mTOR-WT/mTOR*-*kin.def^D2357E,V2364I^; *n =* 3 experiments, unpaired *t* test, Welch-corrected, 2-tailed.*P ≤ 0.05, **P ≤ 0.01.

The identification of the mTORC2 subunit Rictor (17, 18) as the most significant upstream regulator of the altered proteome in *Dyrk1b^–/–^* liver (Figure 6F and Supplemental Table 8) suggested that Dyrk1b may enhance DNL by activating mTORC2. Remarkably, the phosphorylation of mTOR at Ser2448 (p-mTOR-Ser2448) was significantly increased in *Dyrk1b^AAV-WT^* mouse liver versus AAV^control^ (Figure 7A). In contrast, p-mTOR-Ser2448 was significantly reduced in the *Dyrk1b^–/–^* liver (Figure 7B, left panel). The p-mTOR-Ser2481 levels were low and moderately altered in *Dyrk1b^AAV-WT^* (Figure 7A) and *Dyrk1b^–/–^* (Figure 7B, left panel) liver. In vitro studies in malignant cell lines have previously shown that p-mTOR-Ser2448 and p-mTOR-Ser2481 are phosphorylated in mTORC1 and mTORC2, respectively (39, 40). We found that liver p-mTOR-Ser2448 was reduced in mice with hepatocyte-specific knockout of *Rictor* (*LiRctrKO*) (Supplemental Figure 12, A and B) without mTORC1 targets being affected (ref. 17 and Supplemental Figure 12C). In addition, mTOR in the immunoprecipitated mTORC2 from primary WT mouse hepatocytes was phosphorylated at the Ser2448 site, and further increased upon insulin treatment (Supplemental Figure 13A). These findings indicate that mTOR-Ser2448 is phosphorylated in mTORC2 in the liver.

Since Dyrk1b is a serine/threonine kinase, we performed kinase assays to examine whether it can directly phosphorylate mTOR-Ser2448 in either mTORC1 or mTORC2. We confirmed that rhDYRK1B and the immunoprecipitated mTORC1 and mTORC2 retained their enzymatic activities by performing kinase assays with their respective substrates Fkhr, S6K, and Akt (Supplemental Figure 14, A–D). mTORC1 and mTORC2 were immunoprecipitated using anti-Raptor and anti-Rictor antibodies, respectively, using previously described conditions from serum-starved HEK293 cells (ref. 41 and Supplemental Figure 14, E and F). rhDYRK1B robustly catalyzed p-mTOR-Ser2448 in mTORC2 (Figure 7C, row 1, column 2 vs. column 5) but not in mTORC1 (Figure 7C, row 1, column 3 vs. column 6). The p-mTOR-Ser2481 was modestly induced in mTORC2 by Dyrk1b (Figure 7C, row 2, column 2 vs. column 5). Strikingly, *Dyrk1b^AAV-kin.def^* was equally potent in inducing p-mTOR-Ser2448 in mTORC2 compared with *Dyrk1b^AAV-WT^*, indicative of a kinase-independent effect of Dyrk1b (Figure 7D, column 5 vs. column 6; *n =* 4 independent experiments; Supplemental Figure 14G). Furthermore, the Dyrk1b kinase inhibitor AZ191 (42), added in the kinase-wash buffer before and during the kinase reaction at a concentration 1000-fold higher than the IC_50_, failed to inhibit Dyrk1b-dependent p-mTOR-Ser2448 in mTORC2 both using Dyrk1b immunoprecipitates (Figure 7E) and using purified DYRK1B (Figure 7F, row 3). Consistent with the in vitro findings, p-mTOR-Ser2448 was significantly increased by both *Dyrk1b^AAV-WT^* and *Dyrk1b^AAV-kin.def^* in the mouse liver (Supplemental Figure 8C, rows 6 and 7). These data identify mTOR-Ser2448 as a phosphorylation site in mTORC2, triggered by Dyrk1b in a kinase-independent manner.

Next, we investigated whether Dyrk1b could directly activate mTORC2 and whether Dyrk1b-mediated p-mTOR-Ser2448 in mTORC2 is linked to its activation. To this end, we added inactive rhAkt protein 30 minutes after starting the kinase reaction between rhDYRK1B and mTORC2 (Figure 7F). The inactive rhAkt was used to exclude any effects due to enzymatic activity of Akt itself. We observed a dramatic increase in p-AktSer473 upon addition of DYRK1B to mTORC2 (Figure 7F, columns 1–3). Importantly, rhDYRK1B alone did not cause p-AktSer473 (Supplemental Figure 14H), indicating that DYRK1B stimulates p-AktSer473 through activation of mTORC2. AZ191 did not inhibit Dyrk1b-induced activation of mTORC2 (Figure 7F, row 1, columns 3 and 4), indicating kinase-independent activation of mTORC2 by Dyrk1b. The mTOR S2448A point mutation in mTORC2 did not affect Dyrk1b-dependent increase in mTORC2 activity toward p-AktSer473 (Supplemental Figure 13B), suggesting that Ser2448 is a readout of Dyrk1b-dependent mTORC2 activation but does not enhance Akt activation by mTORC2. Altogether, these results indicate that Dyrk1b activates mTORC2 in a kinase-independent manner and this reaction is associated with p-mTOR-Ser2448 in mTORC2.

Since no other kinase, other than mTOR, was present in the described kinase assays, we hypothesized that Dyrk1b stimulates the kinase activity of mTOR itself. Therefore, we used a kinase-defective mTOR in the kinase assay with Dyrk1b. The p-Ser2448 was reduced in kinase-defective mTOR as compared with WT mTOR in the presence of Dyrk1b (Figure 7G, row 1). The canonical p-mTOR-Ser2481 was expectedly reduced in the mTOR kinase-deficient complexes (Figure 7G, row 2). Altogether, these observations indicate that Dyrk1b promotes p-mTOR-Ser2448 by enhancing the catalytic function of mTOR.

Next, we examined the readouts of mTORC1 and mTORC2 activity in the liver. mTORC1 activity, assayed by p-S6K/S6K and p-S6, was higher in *Dyrk1b^AAV-WT^* (Figure 7A) and lower in *Dyrk1b^–/–^* liver (Figure 7B, right panel). p-PKCα/PKCα and p-AktSer473/Akt, the mTORC2 readouts, were increased in *Dyrk1b^AAV-WT^* (Figure 7A) and reduced in *Dyrk1b^–/–^* liver (Figure 7B, right panel). *Dyrk1b^AAV-kin.def^* increased p-PKCα/PKCα, consistent with kinase-independent activation of mTORC2 by Dyrk1b (Supplemental Figure 8C, rows 8 and 9). Taken together, these findings indicate that Dyrk1b directly activates mTORC2, the central regulator of lipogenesis in the liver.

### The hepatocyte-specific disruption of mTORC2 rescues hyperlipidemia, steatosis, and inflammation of Dyrk1b^AAV-WT^ mice.

**Figure 8:**
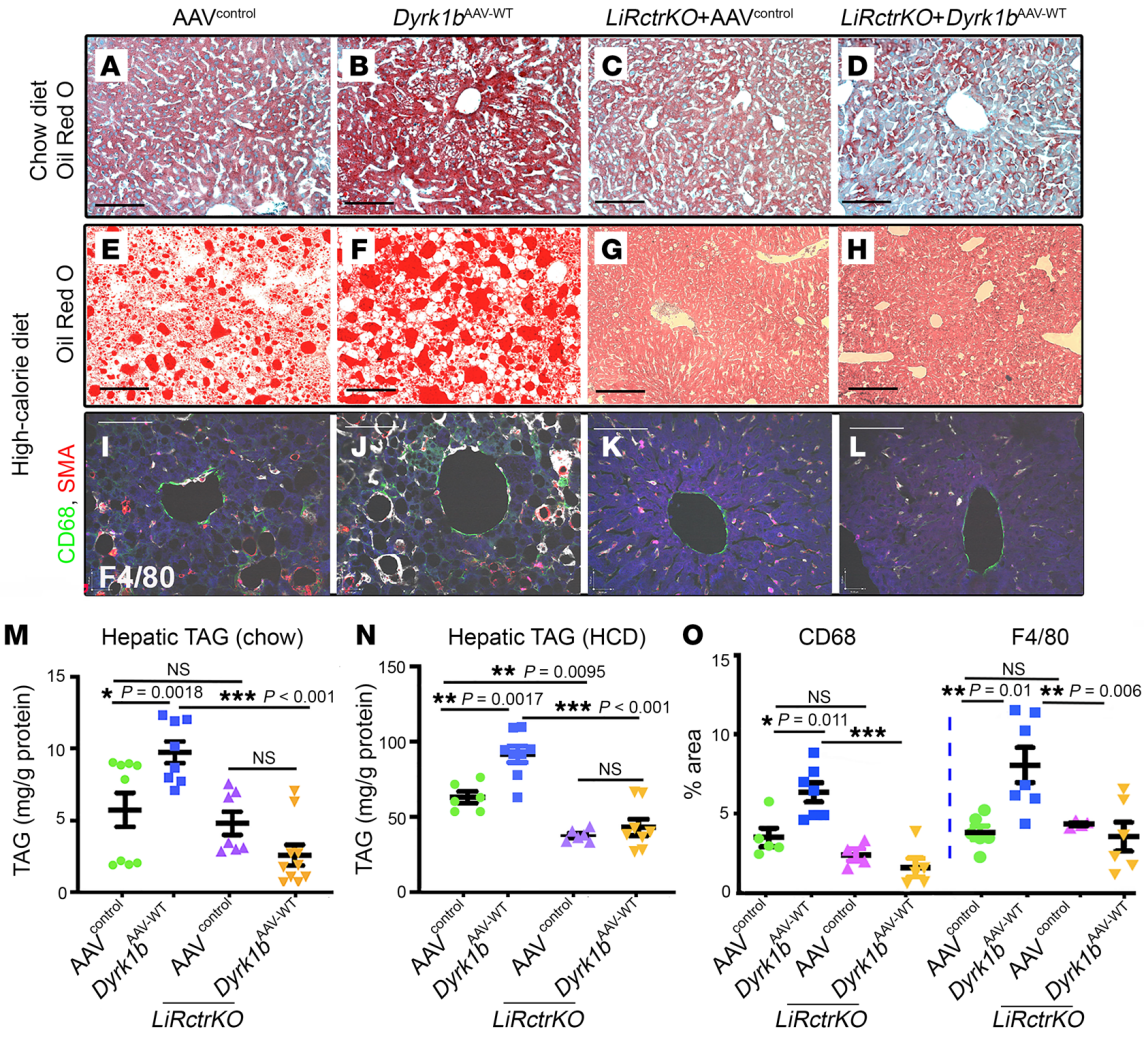
Knockout of *Rictor*, an obligate mTORC2 subunit, rescues steatohepatitis in *Dyrk1b^AAV-WT^* liver. (**A**–**H**) ORO staining in the liver of mice fed with CD (**A**–**D**), *n >* 8 each, or HCD (**E**–**H**), *n >* 6 mice each. (**I**–**L**) CD68 (red), SMA (green), F4/80 (white) costaining in the designated mice fed with HCD for 5 months to induce inflammation; *n >* 6 mice each. (**M**) Hepatic TAG normalized to total protein in the designated mice fed with CD. The data from Figure 2E are repeated to display all the controls together. *n >* 6 mice each, 1-way ANOVA, Tukey’s post hoc test. (**N**) Hepatic TAG normalized to total protein in the indicated genotypes in the mice fed with HCD; *n >* 6 mice each, 1-way ANOVA, Tukey’s post hoc test. (**O**) Quantification of CD68-positive and F4/80-positive area in the designated genotypes by ImageJ; *n >* 5 mice each, 1-way ANOVA, Tukey’s post hoc test. Scale bars: 150 μm. *P ≤ 0.05, **P ≤ 0.01, ***P ≤ 0.001.

**Figure 9:**
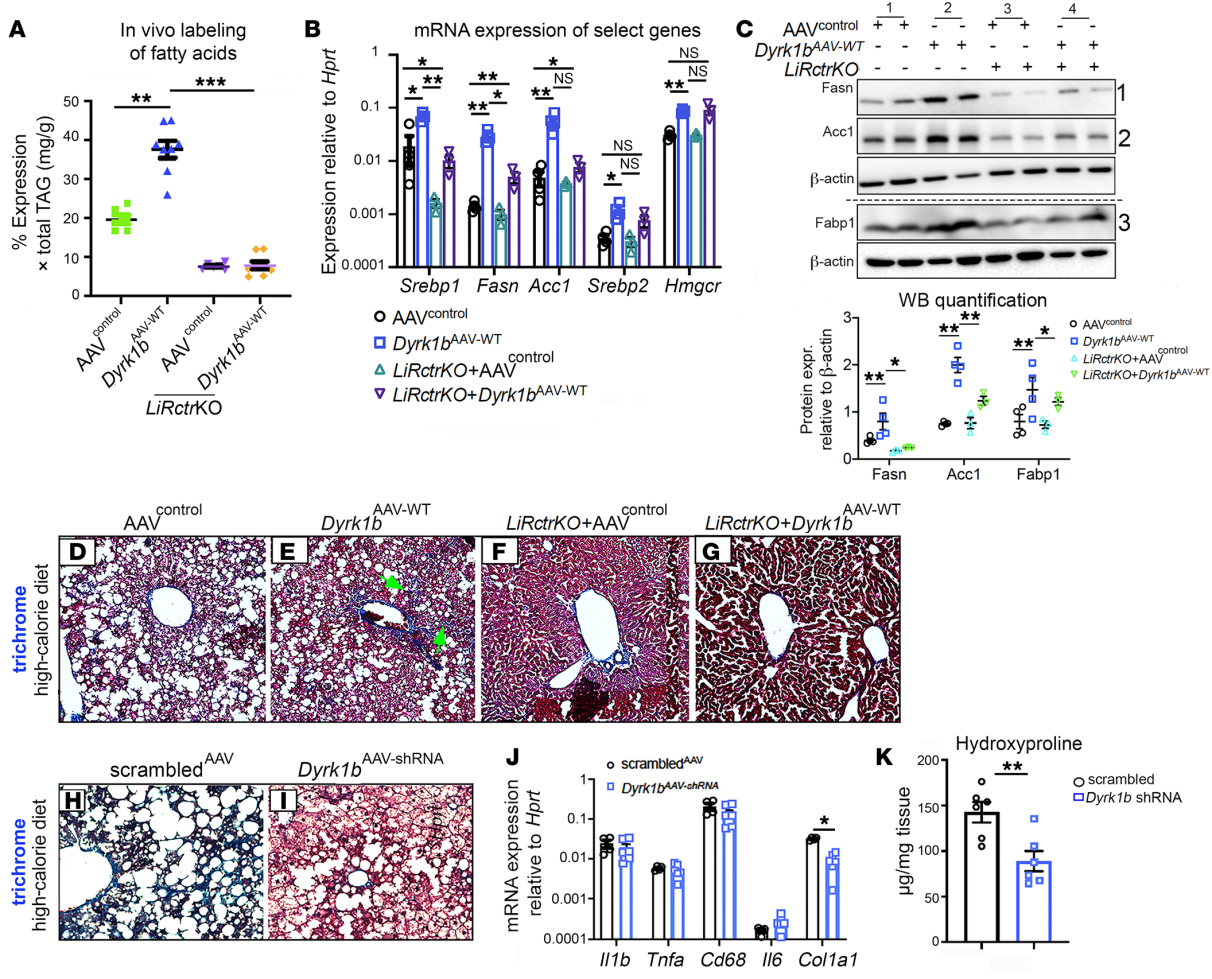
Loss of mTORC2 function rescues DNL and fibrosis in *Dyrk1b^AAV-WT^* liver. (**A**) Isolated palmitate from the liver was quantified for M+1 and M+2 deuterated palmitate and normalized to the control mice given regular water. The data from Figure 4A are repeated to display all the controls together. *n >* 5 mice each, 1-way ANOVA, Tukey’s post hoc test. (**B** and **C**) RNA (**B**) and protein (**C**) expression of selected genes in the DNL pathway in the genotypes indicated. RNA expression was calculated by 2^–ΔΔCt^ method. *n =* 6 mice each group for **B**, *n =* 4 mice for **C**, 1-way ANOVA, Tukey’s post hoc test. (**D**–**I**) Trichrome staining showing collagen (blue) in the liver of mice with designated genotypes. The mice were fed HCD. *n >* 6 mice for each genotype (**D**–**G**); *n =* 4 each (**H** and **I**). Green arrows indicate collagen deposition in the liver sinusoids. (**J**) mRNA expression of the designated genes in scrambled^AAV^ and *Dyrk1b^AAV-shRNA^* mouse liver; *n =* 6 mice each, unpaired *t* test, 2-sided. (**K**) Hydroxyproline levels in the indicated mice; *n =* 6 mice each, unpaired *t* test, 2-sided. *P ≤ 0.05, **P ≤ 0.01, ***P ≤ 0.001.

We next embarked on a genetic rescue study to establish the role of mTORC2 as a mediator of Dyrk1b-induced DNL, hepatic steatosis, and hypertriglyceridemia. We generated mice deficient for hepatic Rictor (*LiRctrKO*), a key component of mTORC2 (17), and injected them and their littermate controls with either empty vector (*LiRctrKO*+AAV^control^) or WT *Dyrk1b* (*LiRctrKO*+*Dyrk1b^AAV-WT^*) and administered CD or HCD for 3 months. The Dyrk1b levels remained unchanged in *LiRctrKO*+*Dyrk1b^AAV-WT^* versus *Dyrk1b^AAV-WT^* mice (Supplemental Figure 2, C and D). Rictor, p-mTOR-Ser2448, and the mTORC2 activity readouts p-AktSer473 and p-PKCα Ser657 were reduced in the *LiRctrKO* liver, while mTORC1 targets remained unchanged (Supplemental Figure 12, A–C). Strikingly, the hepatic steatosis of the *Dyrk1b^AAV-WT^* mice was completely rescued by *LiRctrKO*, on both CD (Figure 8, A–D and M) and HCD (Figure 8, E–H and N). Accordingly, DNL, assayed by deuterium enrichment in palmitate (Figure 9A), and the expression of *Srebp1*, *Fasn*, and *Acc1* were suppressed in *LiRctrKO*+*Dyrk1b^AAV-WT^* (Figure 9, B and C, rows 1 and 2). In addition, plasma TAG was partially, and plasma TC was completely, rescued in *LiRctrKO*+*Dyrk1b^AAV-WT^* mice (Figure 2, F and G). In conclusion, loss of mTORC2 rescues steatosis and hyperlipidemia in *Dyrk1b^AAV-WT^* mice, indicating that Dyrk1b regulates DNL via mTORC2 activation.

We next tested whether alteration of FA uptake by Dyrk1b could be linked to regulation of fatty acid–binding protein 1 (Fabp1), which is regulated by mTORC2 (17). *Fabp1^–/–^* mice were previously reported to have reduced FA uptake in the liver (43). First, we observed that Dyrk1b positively regulated Fabp1 (Figure 9C, row 3, column 2, and Supplemental Figure 9, A and B) in a kinase-dependent manner (Supplemental Figure 8C, row 10). The liver Fabp1 levels were reduced in *LiRctrKO* mice and remained low in *LiRctrKO*+*Dyrk1b^AAV-WT^*, indicating that Dyrk1b regulation of Fabp1 is mTORC2 dependent (Figure 9C, row 3, columns 3 and 4). Additionally, the FA transporter CD36 (platelet glycoprotein 4), but not Fatp1 (solute carrier family 27 fatty acid transporter member 1), was positively regulated by Dyrk1b (Supplemental Figure 9, A–D).

Subsequently, we examined whether Dyrk1b causes inflammation and fibrosis, and whether *LiRctrKO* would rescue these defects. CD68-positive, F4/80-positive macrophages were significantly increased in *Dyrk1b^AAV-WT^* versus AAV^control^ liver (Figure 8, I, J, and O) on HCD, indicating elevated inflammation in the liver. Strikingly, the inflammatory response to *Dyrk1b^AAV-WT^* was completely curtailed in *LiRctrKO*+*Dyrk1b^AAV-WT^* (Figure 8, J–L and O). Notably, the perisinusoidal fibrosis of *Dyrk1b^AAV-WT^* liver, characterized by collagen deposition (Figure 9, D–F, green arrows), was completely rescued by *LiRctrKO* (Figure 9G). *Dyrk1b^AAV-shRNA^* drastically reduced hepatic collagen deposition (Figure 9, H and I), *collagen1a1* expression (Figure 9J), and hydroxyproline levels compared with scrambled^AAV^ (Figure 9K). The expression of proinflammatory cytokines *IL1β*, *IL6*, and *TNFα* remained unchanged in *Dyrk1b^AAV-WT^* (Supplemental Figure 15) and *Dyrk1b^AAV-shRNA^* liver (Figure 9J). The plasma alanine transaminase (ALT) and aspartate aminotransferase (AST) levels also remained unchanged in *Dyrk1b^–/–^*, *Dyrk1b^AAV-shRNA^*, *Dyrk1b^AAV-WT^*, *Dyrk1b^AAV-kin.def^*, and *LiRctrKO* in *Dyrk1b^AAV-WT^* liver (Supplemental Figure 16, A–J). Altogether, these results indicate that mTORC2 mediates the effects of Dyrk1b in induction of DNL, hepatic steatosis, fibrosis, and inflammation.

### Dyrk1b causes insulin resistance by increasing plasma membrane sn-1,2-diacylglycerol, leading to translocation of PKCε and reduced IRK activity.

**Figure 10:**
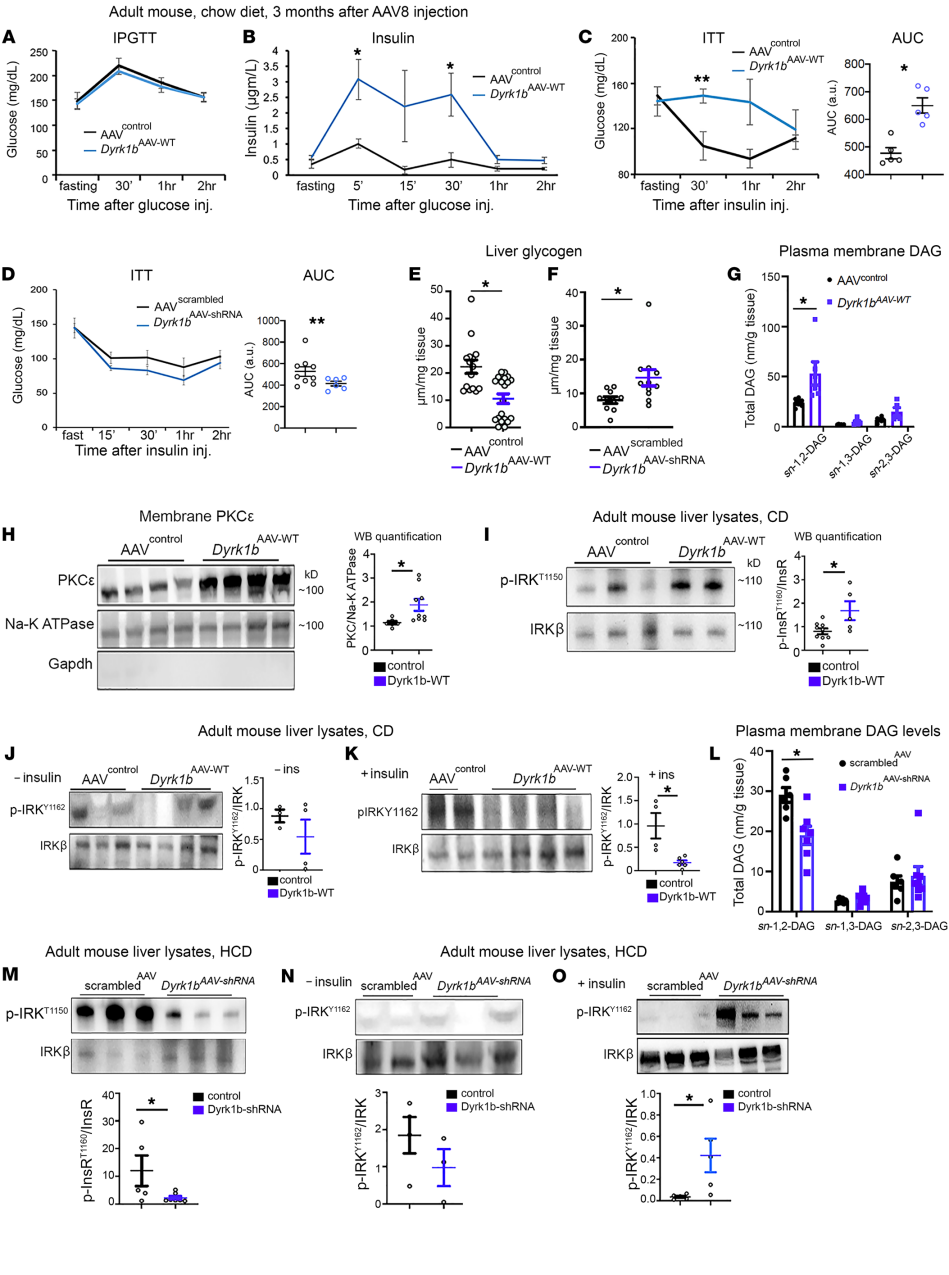
Dyrk1b increases membrane DAG and impairs insulin receptor activation. (**A** and **B**) Glucose (**A**) and insulin (**B**) during intraperitoneal glucose tolerance test (IPGTT) in *Dyrk1b^AAV-WT^* mice after 6-hour fast; *n >* 8 mice per group, 1-way ANOVA, Holm-Šidák post hoc test, *P* (area under the curve) = 0.0025. (**C** and **D**) Insulin tolerance test (ITT) on *Dyrk1b^AAV-WT^* (**C**) and *Dyrk1b^AAV-shRNA^* mice (**D**). After 6 hours of fasting, fast-acting Humulin (0.75 U/kg) was injected i.p., and glucose was measured at indicated time points. *n >* 8 mice per group, 1-way ANOVA, Holm-Šidák post hoc test. (**E** and **F**) Liver glycogen quantification in the indicated mice after fast/refeed for 6 hours; *n >* 13 mice each group for **E**, *n >* 9 for **F**, unpaired *t* test, 2-sided. (**G**) Plasma membrane *sn*-1,2-, *sn*-1,3-, and *sn*-2,3-DAG levels in *Dyrk1b^AAV-WT^* mice; *n >* 6 mice each group, unpaired *t* test, 2-sided. (**H**) Membrane PKCε levels after fractionation into membrane and cytosolic fractions; *n >* 6 mice each group, unpaired *t* test, 2-sided. (**I**) p-IRK^T1150^ enrichment by immunoprecipitation in liver lysates of the indicated mice; *n >* 4 mice each group, unpaired *t* test, 2-sided. (**J** and **K**) p-IRK^Y1162^ in liver samples from *Dyrk1b^AAV-WT^* mice only fasted (**J**) or fasted and stimulated with insulin for 15 minutes (**K**); *n >* 3 mice each group, unpaired *t* test, 2-sided. (**L**) Plasma membrane *sn*-1,2-, *sn*-1,3-, and *sn*-2,3-DAG levels in *Dyrk1b^AAV-shRNA^* mice; *n >* 6 mice each, unpaired *t* test, 2-sided. (**M**) p-IRK^T1150^ enrichment by immunoprecipitation in the indicated liver lysates; *n >* 4 mice each group, unpaired *t* test, 2-sided. (**N** and **O**) p-IRK^Y1162^ in liver samples from fasted (**N**, *n =* 3 mice each group) and insulin-stimulated (**O**, *n >* 5 mice each group) *Dyrk1b^AAV-shRNA^* mice; unpaired *t* test, 2-sided. *P ≤ 0.05, **P ≤ 0.01.

We next investigated whether Dyrk1b causes hepatic insulin resistance (IR), commonly associated with NAFLD and T2D (44). The measurement of global glucose homeostasis with an intraperitoneal glucose tolerance test showed unchanged plasma glucose levels while the plasma insulin levels were elevated in *Dyrk1b^AAV-WT^* mice compared with AAV^control^, indicating whole-body IR (Figure 10, A and B). The insulin tolerance test confirmed resistance to insulin action in *Dyrk1b^AAV-WT^* versus AAV^control^ (Figure 10C) and conversely increased insulin sensitivity in *Dyrk1b^AAV-shRNA^* (Figure 10D). Accordingly, the hepatic glycogen stores were significantly reduced in *Dyrk1b^AAV-WT^* and were increased in *Dyrk1b^AAV-shRNA^* mouse liver (Figure 10, E and F). Earlier studies had shown that increased FA flux could result in increased plasma membrane *sn*-1,2-diacylglycerol (*sn*-1,2-DAG), which then promotes hepatic IR through increased translocation of PKCε to the plasma membrane, resulting in increased inhibitory modification of insulin receptor kinase (IRK) by T1150 phosphorylation (IRK^T1150^) (7, 8). We therefore examined whether increased DNL and FA uptake in *Dyrk1b^AAV-WT^* liver is associated with increased plasma membrane *sn*-1,2-DAG and its associated molecular changes. As predicted, the liquid chromatography–tandem mass spectrometry analysis showed increased plasma membrane *sn*-1,2-DAG levels in the liver lysates from *Dyrk1b^AAV-WT^* versus AAV^control^ (Figure 10G). The higher levels of plasma membrane *sn*-1,2-DAGs were associated with increased PKCε translocation to the plasma membrane (Figure 10H) and increased IRK^T1150^ phosphorylation (Figure 10I) in *Dyrk1b^AAV-WT^* versus AAV^control^ liver. While hepatic p-IRK^Y1162^ was not detectable in fasted mice (Figure 10J), it was increased upon insulin stimulation in AAV^control^ but not *Dyrk1b^AAV-WT^* mice (Figure 10K, and Supplemental Figure 17A for additional replicates), indicating impaired IR in the latter. Conversely, in *Dyrk1b^AAV-shRNA^* mouse liver, the plasma membrane *sn*-1,2-DAG levels (Figure 10L) and IRK^T1150^ phosphorylation (Figure 10M) were decreased and p-IRK^Y1162^ was increased upon insulin stimulation (Figure 10, N and O, and Supplemental Figure 17B for additional replicates). Taken together, these results indicate that Dyrk1b causes liver IR by inducing plasma membrane *sn*-1,2-DAG/PKCε–dependent impairment of IRK activity, while its suppression protects against diet-induced hepatic IR.

## Discussion

The identification of *Dyrk1b* as a disease gene for abdominal obesity–metabolic syndrome (OMIM AOMS3) provided an exceptional opportunity to explore the underlying disease mechanisms and for the discovery of drug targets for a disorder that has one of the fastest growing incidences. In this article, we have characterized the function of Dyrk1b in a number of transgenic mouse models and have made several paradigm-shifting discoveries. First, we show that Dyrk1b is a relevant drug target not only for those with mutations in the gene but for most patients with NASH, in whom its levels were found to be considerably elevated. Secondly, increased expression of Dyrk1b is sufficient for the development of hyperlipidemia, fatty liver disease, liver inflammation, and fibrosis on CD. Most relevantly, we discovered that the knockdown of hepatic *Dyrk1b* protects against diet-induced hepatic IR and NASH and, hence, is an attractive drug target.

At the molecular level, we have discovered metabolic pathways by which Dyrk1b induces hepatic steatosis (a) by activating mTORC2 and augmenting DNL and (b) by upregulating Fabp1 and CD36 and increasing FA influx into the liver. These in turn lead to increased FA-CoA pool, esterification of FAs into DAGs and TAGs, PKCε activation, and IRK^T1150^ phosphorylation and IRK inactivation. The reduced IRK activity is the basis for DAG-induced IR as previously described (7, 8). Concisely, Dyrk1b increases DNL and FA uptake in an insulin-resistant liver by bypassing the canonical insulin signaling and directly activating mTORC2. Although induction of DNL by Dyrk1b is mediated by mTORC2, targeting *Dyrk1b*, but not mTORC2, has potential advantages for the treatment of NASH. First, the inhibition of mTORC2 causes IR while *Dyrk1b* knockdown is protective against IR. Further, disruption of *Dyrk1b* reduces hepatic FA uptake, leading to reduced DAG and TAG. In an insulin-resistant state, the augmentation of both hepatic FA uptake (65%) and DNL (25%) contributes to hepatic steatosis in human NAFLD (34, 45). Interestingly, the DNL rates have been shown to be increased 5-fold in individuals with obesity (46) and T2D (47) and up to 3-fold in individuals with NAFLD compared with healthy controls (48). Interestingly, mice carrying *Dyrk1b^R102C^* mutation recently generated in the laboratory exhibit increased *Drk1b* mRNA levels (our unpublished observations). One may extrapolate these data to human DYRK1B^R102C^ carriers, in whom increased liver DYRK1B may cause NAFLD and IR. An extensive characterization of *Dyrk1b^R102C^* mice may provide further insight into the pathogenesis of MetS in the mutation carriers. Taken together, these findings underscore the role of Dyrk1b as an attractive therapeutic target for the treatment of both NAFLD and T2D in the general population and for human DYRK1B^R102C^ carriers.

The AAV8-mediated gene therapy is a promising method to target the liver as demonstrated by clinical trials for various morbidities. We verified that, with the use of AAV8, Dyrk1b was changing specifically in the liver and not in other metabolic organs that affect global glucose and triglyceride homeostasis such as adipose tissue, skeletal muscle, and pancreas. Importantly, we rescued the effects on plasma TAG, TC, and hepatic TAG in *Dyrk1b^AAV-WT^* with hepatocyte-specific deletion of mTORC2 function. Nevertheless, the potential contribution of other liver cell types to metabolic traits and specifically liver inflammation and fibrosis remains to be determined.

The activation of mTORC2 by Dyrk1b was associated with increased p-mTOR-Ser2448. p-mTOR-Ser2448 was previously associated with mTORC1 activation in cancer cell lines (39). In contrast, we detected p-mTOR-Ser2448 in mTORC2 in mouse hepatocytes, a distinction that may be due to cell-specific effects. In addition, p-mTOR-Ser2448 was dramatically reduced in *LiRctrKO* mouse liver despite no differences in mTORC1 readouts (Supplemental Figure 12). Although p-mTOR-Ser2448 did not alter mTORC2 activation toward Akt, it was always associated with the activated state of mTORC2 in different transgenic mouse models. Whether Dyrk1b-mediated p-mTOR-Ser2448 alters the function of other yet unknown targets of mTORC2 remains to be determined. Also of interest is the potential interaction between nuclear Dyrk1b and nuclear mTOR, which affects transcription from several metabolic genes, including *IRK*, *IRS1/2*, *Gsk3b*, *G6pc*, and *LPL* (49). Finally, we noted a Dyrk1b-dependent increase in p-mTOR-Ser2448 in the kinase assays with mTORC2 but not mTORC1. The activation of mTORC1 by Dyrk1b appears to be indirect, as Dyrk1b failed to increase p-mTOR-Ser2448 in mTORC1 or increase p-S6K in the in vitro kinase assays (data not shown). Despite no direct effects on mTORC1, Dyrk1b did increase mTORC1 activity in vivo. Since disruption of mTORC2 almost completely rescued DNL in the *Dyrk1b^AAV-WT^* model, the contribution of mTORC1 toward DNL is probably incremental. The mTORC1 activation may, however, contribute to IR in *Dyrk1b^AAV-WT^* mice (15, 14) and complement the effect of DAG-induced IRK inhibition. The consequences of Dyrk1b activation of mTORC1 are beyond the scope of the current study and are a topic of future investigations.

In conclusion, our findings demonstrate that Dyrk1b is highly expressed in NASH, activates mTORC2, and causes hypertriglyceridemia, fatty liver, and hepatic IR. Considering the multifaceted role of mTOR in many disease pathways, these findings have implications that go beyond pathway discovery for NASH and are relevant for understanding the pathogenesis of many other mTOR-regulated disease processes, such as malignancies, aging, and neurodegenerative diseases (11).

## Methods

### Study design.

For every experimental condition, littermate controls were used and subjected to identical experimental conditions as specified. The investigators were not blinded to the animal genotypes. For all immunoprecipitation/kinase reactions, the control and experimental reactions were carried out in the same batch of cells and processed together. The human biopsies for Dyrk1b expression studies were randomly selected from the Yale Repository and diagnosed for NASH by the pathologists of Yale New Haven Hospital. The samples were deidentified, and the medical information of the patients was accessed through the medical record number by designated personnel.

### Mouse colony maintenance, diet, and genotyping.

The mice were housed at a constant temperature in a 12-hour light/12-hour dark cycle and were fed ad libitum with the indicated diets: HCD (Research Diets, D12108C), high-fat diet (Research Diets, D12492), high-sucrose diet (Research Diets, D12450B). For genotyping, the mice were ear-tagged, and DNA was extracted (Qiagen, 69506) and genotyped by optimized PCR protocols or as specified by The Jackson Laboratory. *Alb:Cre* and *Rictor^fl/fl^* mice were obtained from The Jackson Laboratory.

### Cryosectioning, immunofluorescence, and quantification.

The liver samples were processed by standard procedures for immunofluorescence (50) and imaged by SP8 confocal or 4-laser scanning confocal microscope. The following antibodies were used: F4/80 primary (Abcam, ab6640, lot GR3189625-1), secondary (Abcam, ab150155); SMA primary (Abcam, ab7817, lot GR43049-2), secondary (Thermo Fisher Scientific, A28175); CD68 PE-conjugated (BioLegend, 137013, lot 13192561). For Dyrk1b immunofluorescence, antigen retrieval was performed in 10 mM sodium citrate buffer, pH 6, 0.05% Triton according to Thermo Fisher Scientific, B40922. The paraffin-coated human biopsies were deparaffinized (50). The Dyrk1b antibodies for immunofluorescence were: primary (Abcam, ab124960, lot GR82993-10), peptide primary (Abcam, ab246843, lot GR3268737), secondary (Invitrogen, 31460). The fluorescent reaction was developed using Tyramide signal amplification (Thermo Fisher Scientific, B40922). For preblocking antibodies, 5-fold excess amount of peptide was incubated with the antibody overnight at 4°C. The quantification was done using ImageJ (NIH) by analysis of particles obtained after color thresholding.

### Blood collection and plasma TAG, TC, LPL, and NEFA measurements.

The animals were fasted for 6 hours starting at 9 am and anesthetized in 30% isoflurane, and 100 μL of blood withdrawn by retro-orbital bleeding into heparin-coated tubes (Minicollect, 450477). The blood was spun down at maximum speed for 15 minutes at 4°C, and plasma was frozen. TAG was measured by Fujifilm Wako Diagnostics (catalog 994-02891, 990-02991), TC by a Cell Biolabs assay kit (catalog STA-384), and NEFA by an Abcam assay kit (catalog ab65341).

### Oil Red O staining.

For Oil Red O staining, the manufacturer’s guidelines (Abcam, ab150678) were followed and slides mounted in aqueous media (Sigma-Aldrich, GG1).

*AAV virus production and i.p*. *injection into mice*. The AAV-DJ/8 Helper Free Expression System (Cell Biolabs, VPK-410-DJ-8) was used to generate the virus. The N-terminal FLAG-tagged Human DYRK1B ORF (Sino Biologicals, HG12248-NF) and DYRK1B^K140R,Y273F^ were cloned into the AAV expression vector. The plasmids encoding adenoviral gene products and Rep-Cap proteins and AAV expression vectors encoding DYRK1B-WT or DYRK1B^K140R,Y273F^ or with no insert (AAV control) were cotransfected into 293AAV cells (Cell Biolabs, AAV-100). The virus was released from the cell lysates according to the manufacturer’s instructions (AAV-DJ/8 Helper Free Expression System), and nucleic acids were digested by benzonase (50 U/mL) at 37°C for 30 minutes and purified by iodixanol density-gradient ultracentrifugation. The titers of AAV were obtained by quantitative PCR (qPCR; Clontech, 632252) using the following primers: forward, AGTTGGCCACTCCCTCTCTGC; reverse, TGCAGGCAGCTGCGCGCT. One hundred vector genomes were injected i.p. per mouse twice at a duration of 1 month, and mice were sacrificed 3 months later. AAV8-GFP (GFP in the expression vector) was injected to check for in vivo transduction efficiency.

### Hepatocyte culture and AAV8 transduction.

The hepatocytes were prepared by standard procedures at Yale Liver Center, spun down at 50*g*, plated on collagen-coated dishes at a density of 10^5^/cm^2^, and maintained in William’s E media plus 10% FBS plus 1× supplement (Thermo Fisher Scientific, CM4000) plus 1 μM dexamethasone in a 5% CO_2_ incubator. For in vitro transduction experiments, the titrated AAV8 viral particles were transduced at an MOI of 60 in William’s E media plus 1 nM insulin plus 1 μM dexamethasone for 24 hours. Seventy-two hours after transduction, cells were fixed in 10% formalin for Oil Red O staining.

*Seahorse experiments for determining β*-*oxidation of fatty acids*. The β-oxidation assay was carried out with the Seahorse XF Cell Mito Stress Test Kit per the manufacturer’s instructions (Agilent, part 02720-100) in the 96-well format with hepatocyte density of 2 × 10^3^ cells per 100 μL. The Mito Stress Test (oligomycin 2.5 μg/mL, FCCP 0.8 μg/mL, 2 μM rotenone) was performed under the following 4 conditions: BSA, BSA + 400 μM etomoxir, BSA-palmitate, and BSA-palmitate + 400 μM etomoxir. For virus transduction experiments, the cells were incubated in AAV8 in William’s E media plus 1 nM insulin plus 1 μM dexamethasone, for 24 hours before the assay.

### Hepatic TAG quantification.

The liver was processed according to the protocol described in ref. 51 and TAG quantified by Fujifilm Wako Diagnostics (catalog 994-02891, 990-02991) and normalized to total protein content, estimated by bicinchoninic acid (BCA) assay.

### Dyrk1b AAV-shRNA production.

The plasmids containing scrambled shRNA and 4 *Dyrk1b* shRNAs were purchased from Origene Inc. (catalog TL514285) and shuttled into the AAV vector with U6 promoter and inverse terminal repeats (Origene Inc., TR30034). The sequences of the *Dyrk1b* shRNAs and scrambled shRNA are reported in Supplemental Table 2. The viruses were prepared, purified, and titrated as described above (Cell Biolabs, VPK-410-DJ-8). The viruses containing each of the 4 shRNAs were combined in equal amounts and coinjected at the titer of 4 × 10^9^ viral genomes per mouse. AAV particles with scrambled shRNA were injected at identical titers.

### Generation of Dyrk1b homozygous mice.

A gene trap vector containing a cryptic splice acceptor site at the 5′ end to the neomycin-polyA sequence was designed to target intron 2 of the endogenous *Dyrk1b* gene. C57BL/6 embryonic stem cells were injected with the constructs and targeted into the BALB/c blasts to generate chimeras. Southern blot analysis on a limited number of chimeric embryos confirmed the integration of the modified DNA. Chimeras were backcrossed for 7 generations with B6 mice. The mice from different chimeras gave identical phenotypes. The viable offspring were genotyped by PCR using the following primers: wild type, forward ATGAGAATAAGCTGAGACCAGG, reverse AGCTGACGACTCACTGACCATG; *loxP1*, forward GGGGTGGAAATGAGAATAAGC, reverse TACCGGTGGATGTGGAATGT; *loxP2*, forward CCCCCTGAACCTGAAACATA, reverse GGAGACCCAAGCTGGCTAGT.

### Kinase assays.

The immunoprecipitation of endogenous mTORC2 and mTORC1 was done from the HEK293 cells, obtained from American Type Culture Collection. The cells were cultured in 10% FBS, 1× penicillin-streptomycin, and 4.5 g/L glucose. Each 10 cm culture dish was transfected with the empty vector, *Dyrk1b^WT^*, or *Dyrk1b^K140R,Y273F^*. The cells were serum-starved overnight in 1 g/L glucose DMEM and harvested in the lysis buffer (40 mM HEPES, 10 mM glycerobeta-phosphate, 0.3% CHAPS, 10 mM sodium pyrophosphate, 1 mM EDTA, 100 mM NaCl) (41, 52). The lysates were rocked for 10 minutes at 4°C, followed by centrifugation at 18,928*g* for 10 minutes. The protein concentration was estimated by BCA assay (Pierce, 23225) and adjusted to the same concentration across different lysates. Two milligrams of lysate was used for each immunoprecipitate containing 1 μg of the antibody, and incubated for 2 hours at 4°C: IgG (ImmunoReagents, GtxRb-003-E3, lot 47-180-042817), anti-Rictor (Bethyl Labs, A300-459A, lot 4), and anti-FLAG antibody (MilliporeSigma, F3165). Subsequently, protein A/G agarose beads (Pierce, 20421, lot TB261674) were added only to the lysates containing the FLAG antibodies for 1 hour at 4°C. The agarose beads were centrifuged at 4032*g* for 30 seconds, and the supernatant was discarded. To each of the immunoprecipitation reactions from empty vector–transfected cells, either IgG or mTORC2 (immunoprecipitated with anti-Rictor antibody) was added. Similarly, to each of the lysates from *Dyrk1b^WT^-* and *Dyrk1b^K140R,Y273F^*-transfected cells, complexes containing IgG or anti-Rictor antibody were added for 1 hour at 4°C. This was done to capture FLAG-tagged Dyrk1b and mTOR complexes onto the same beads. The beads were washed 4 times for 5 minutes with 1 mL of lysis buffer at 4°C.The last wash was done in 1 mL of 1× kinase reaction buffer (KRB; 25 mM HEPES, 100 mM potassium acetate, 1 mM MgCl_2_) for 5 minutes at 4°C. The kinase reaction was carried out in 15 μL of KRB along with 500 μM ATP, for 30 minutes at 37°C, and stopped by addition of 50 μL of 2× Laemmli buffer. All incubations at 4°C were done on an end-to-end rotator. For the kinase reactions involving purified proteins, the immunoprecipitated mTORC2 or IgG was incubated with purified recombinant human DYRK1B (rhDYRK1B; Creative BioMart, DYRK1B-2983H, 81% pure) in the KRB buffer plus 500 μM ATP.

To pharmacologically inhibit DYRK1B, the rhDYRK1B was preincubated in either DMSO or AZ191 at a concentration of 10 μM for 10 minutes at room temperature. DMSO or AZ191 was added in KRB during washes and during the kinase reactions. The following purified proteins were used: rhFkhr (MilliporeSigma, 14-343, >40% pure), rhAkt (MilliporeSigma, inactive, 14-279-D, 67.7% pure), S6K (R&D Systems, 896-KS, >70% pure).

For mTORC2 activity assay, a kinase reaction was carried out between rhDYRK1B or Dyrk1b immunoprecipitate and mTORC2 as described above in the presence of 500 μM ATP for 30 minutes at 37°C. Subsequently, rhAkt was added to the reactions for another 30 minutes. The reaction was stopped by 2× Laemmli buffer.

### Coimmunoprecipitation assays.

The primary hepatocytes from WT C57BL/6J mice were plated as described above. The cells were collected after either overnight serum starvation or 15 minutes of insulin (MilliporeSigma, 10516) treatment after serum starvation.

### Fatty acid uptake experiment.

The fatty acid uptake experiment was conducted as described in ref. 53. Briefly, hepatocytes were serum-starved overnight followed by insulin stimulation and addition of BODIPY-C16FL (2 μM Thermo Fisher Scientific, D3821) diluted in HBSS plus 10% delipidated serum; fixed in 10% formalin; and imaged by confocal microscopy.

### Labeling of de novo synthesized triglycerides in mice and purification of palmitate from liver.

The mice were fed a sucrose diet (Research Diets, D12450B) for 1 week before being fed deuterium oxide (D_2_O). After 7 days, the mice were injected i.p. with 23.4 mL/kg body weight filter-purified D_2_O solution (Cambridge Isotope Labs, DLM-4-100) and kept on the same sucrose-rich diet and water containing 5% D_2_O for another 7 days. The mice were subsequently sacrificed, the liver tissue snap-frozen at –80°C, and plasma collected. Hepatic triglycerides were prepared using the method of Folch et al. (54). Briefly, 100 mg of liver tissue was homogenized in precooled 2:1 chloroform/methanol solution, with stainless steel beads (Next advance, SSB14B), followed by incubation for 3–4 hours at room temperature. Next, bottom organic phase was recovered after addition of 0.1 M H_2_SO_4_. The palmitate was isolated by thin-layer chromatography (Agilent, TLC Silica, HX71211115) and separated in 80% hexane plus 20% diethylether and 0.01% glacial acetic acid. The plate was sprayed with primuline solution (MilliporeSigma, 206865; 80% acetone, 20% water, a pinch of primuline), and palmitate visualized under UV light and subsequently scraped from the TLC plate. The palmitate was extracted by diethylether, dried in nitrogen, converted into its methyl-ester derivative by heating at 70°C for 45 minutes in boron trifluoride (MilliporeSigma, B1252), extracted in pentane, dried in nitrogen, and subjected to gas chromatography–mass spectrometry analysis. All values were normalized to the plasma D_2_O, and to palmitate from control mice not given D_2_O water.

### VLDL secretion by poloxamer-407.

The protocol for measurement of VLDL secretion by poloxamer-407 was performed as detailed in ref. 51.

### Metabolic cages.

The metabolic cage studies were done at Yale Mouse Metabolic Phenotyping Center.

### Generation of kinase-defective Dyrk1b and mTOR Ser2448A.

The primers with mutated residues were designed with about 20 bp flanking either side of the mutation and PAGE-purified. The plasmids were constructed using Agilent QuikChange (catalog 200521) and sequenced in their entirety: K140R, forward GACCCAGGAGCTTGTGGCCATCAGAATCATCAAGAACAAAAAGGC, reverse GCCTTTTTGTTCTTGATGATTCTGATGGCCACAAGCTCCTGGGTC; Y273F, forward TGGCCAGAGGATCTACCAGTTCATCCAGAGCCGCTTCTACCG, reverse CGGTAGAAGCGGCTCTGGATGAACTGGTAGATCCTCTGGCC. For generating point mutation in mTOR, the In-Fusion cloning method (Takara Biosciences) was used, with the following primers: forward, GGACGGATGCCTACTCTGCTGGCCAGTCAGTC; reverse, AGTAGGCATCCGTCCTCGTTCGGGATC.

### Western blotting and quantification.

Western blotting was done using standard procedures, and the membrane was developed by SuperSignal West Pico (Thermo Fisher Scientific, 34580) and SuperSignal West Femto (Thermo Fisher Scientific, 34096). The Western blots were quantified by ImageJ and the heatmaps generated by GraphPad Prism 8.0. The following antibodies were used at 1 μg/mL, unless otherwise noted: Fasn (Cell Signaling Technology [CST], 3189S, lot 2), Acc (CST, 3662S, lot 4), F4/80 (Abcam, ab6640, lot GR3189625-1), Akt2 (CST, 2962, lot 2; CST, 9272S, lot 25), Dyrk1b (1:500; Abcam, ab124960, lot GR82993-10; Novus Biologicals, NBP1-33464, lot 40135), Fabp1 (CST, 5352S, lot 1), p-AktSer473 (CST, 9271S, lot 14), β-actin–HRP (CST, 5125S, lot 6), p-mTOR-Ser2448 (CST, 2971S, lots 22, 27), mTOR (CST, 2972S, lot 10), p-IRS-Tyr608 (EMD Millipore, 09-432, lot 2876222), HMGCR (Invitrogen, PA5-37367, lot SD2371035A), p-S6 (CST, 2215S, lot 14), S6 (CST, 2217S, lot 5), p-p70S6K-Thr389 (CST, 9205S, lot 21), PKCα (Invitrogen, PA5-13739, lot UA2699692), p70S6K (CST, 2708S, lot 7), p-PKCα Ser657 (Millipore, 06-822, lot 2332540), SMA (Abcam, ab7817, lot GR43049-2), Rictor (Bethyl Labs, A300-459A, lot 4), Raptor (Bethyl Labs, A300-553A), HMGCS1 (Bethyl Labs, A304-590A), p-Akt Thr308 (CST, 9275S, lot 20), p-mTOR-Ser2481 (EMD Millipore, 09-343, lot 2549367), Srebp1 (Santa Cruz Biotechnology, H-160, sc-8984, lot L1509), Srebp2 (Santa Cruz Biotechnology, C-6, sc-271615, lot A1711), CD68 (BioLegend, 137013, lot B192561), FLAG (MilliporeSigma, F1804, lot SLBV9325), G6pase1 (Novus Biologicals, NBP1-80533, lot QC58207), Fatp1 (BIOSYS, B59454R, lot 9C24M37), CD36 (Novus Biologicals, NB400-144SS), MTTP (Abcam, ab75316, lot GR204405-6), ApoB (Santa Cruz Biotechnology, SC393636, lot B0921), Pten (CST, 9188).

### RNA extraction, cDNA synthesis, and qPCR.

The RNA was extracted using a Qiagen RNeasy Mini Kit (catalog 74104), treated with DNase (Promega, M6106), and repurified to remove DNase. One microgram of RNA was used for cDNA synthesis (Bio-Rad, 1708890). No–reverse transcriptase and no-RNA controls were included for each sample. The qPCR was performed with 2× SYBR Green Master Mix (Bio-Rad), 0.5 μM primer (forward plus reverse), and diluted cDNA. qPCR was carried out with *n =* 3 technical replicates and at least 6 biological replicates per genotype. All values were normalized to the reference genes *Hprt* and *β-actin*. The following primers were used: *Dyrk1b*, forward TAGAGCGCTATGAGATTGACTCTC, reverse TAGCTCAATCTGTGCCTGGTTCA; *Srebp1*, forward TGACCCGGCTATTCCGTGA, reverse CTGGGCTGAGCAATACAGTT; *Srebp2*, forward TTCCAACTCTCCTCCTGTGGCT, reverse CCAGCACAAATAAGCAGGTTTGTA; *Fasn*, forward GGAGTTCTCAGGCCGGGATA, reverse GGGTACATCCCAGAGGAAGTCA; *Acc1*, forward TGGGGATCTCTGGCTTACAGG, reverse AGCCAGACATGCTGGATCTCAT; *Hmgcr*, forward AAGACTGTGGTTTGTGAAGCCGTCA, reverse TTGTAGCCGCCTATGCTCCCAG; *Hprt*, forward TGTTGTTGGATATGCCCTTG, reverse GCGCTCATCTTAGGCTTTGT. The primers for *IL1β*, *TNFα*, *IL6*, and *Col1a1* were reported previously (50).

### Proteomics.

The tissues were processed as described in ref. 55. Three independent samples were analyzed for proteomics analysis for each genotype. The fold changes corresponding to each protein were calculated and subjected to the following filters: unique peptides >2, *P* value ≤0.05, 2-tailed unpaired Student’s *t* test, FDR ≤0.05, Benjamini-Hochberg correction. The hierarchical clustering of the variables and the heatmaps for differentially expressed proteins were created by Qlucore Omics Explorer 3.4.

### Glycogen extraction.

Glycogen extraction was performed with a Sigma-Aldrich Glycogen Assay Kit (MAK016) according to the manufacturer’s guidelines.

### PKCε translocation and IRK^T1150^ phosphorylation.

PKC*ε* translocation and IRK^T1150^ phosphorylation were done as described previously (8).

### Plasma alanine transaminase and aspartate aminotransferase.

Plasma alanine transaminase and aspartate aminotransferase were measured with Colorimetric Activity Assay Kits (Cayman Chemicals, 700260, 701640) per the manufacturer’s protocol.

### Hydroxyproline measurement.

The hydroxyproline measurement protocol was as described by Cell Biolabs, catalog STA-675. Briefly, liver tissues were homogenized in water, and an aliquot was kept for protein estimation. The samples were acid-hydrolyzed for 24 hours in 12N HCl, cleared by activated charcoal, and centrifuged at 10,000*g* for 5 minutes. A 10 μL aliquot was dried at –80°C and hydroxyproline estimated by absorbance at 540 nm after addition of chloramine T and Ehrlich’s reagent.

### Statistics.

The comparisons between 2 groups were done by Student’s *t* test, and multiple-group comparisons were done by 1-way ANOVA with Tukey’s post hoc test or 1-way ANOVA with post hoc Holm-Šidák test. Normality was tested by Kolmogorov-Smirnov test, with >0.1 considered normal. If the data were not normal, nonparametric Mann-Whitney *U* test was used as specified in the text. If the differences in the variances between samples were found to be significant, the *P* values were Welch-corrected. Each dot in the dot plots and each lane of Western blots represent a biological replicate. The values in the graphs represent average ± SEM, unless otherwise stated. The following thresholds were used for statistical significance: *P* ≤ 0.05, *P* ≤ 0.01, *P* ≤ 0.001. For area under the curve, Student’s *t* test was used to calculate significance. About 7–10 animals were used per genotype, as specified in the text. For hepatocyte cultures, each experiment was done in triplicate from 3 sets of mice, unless otherwise stated. All statistical tests were performed with GraphPad Prism 8.0.

### Study approval.

The animal research was carried out according to a protocol approved and overseen by the Institutional Animal Care and Use Committee of Yale University. The study of human clinical samples was conducted in compliance with the provisions of the Declaration of Helsinki, and the study protocol was approved by the institutional review board of Yale University. All subjects gave informed consent for the use of their biopsies for scientific purposes.

## Author contributions

NB designed and performed experiments, generated constructs, and prepared figures. A Narayanan contributed to study design. MF assisted with creation and genotyping of *Dyrk1b^–/–^* mice. MK performed *sn-*1,2-DAG quantification. DZ and LG assisted with protocols. A Neogi prepared demographics of NASH patients. RC and RK performed and interpreted Seahorse studies. RK supervised Seahorse studies. CFH provided liver samples from mice on a high-fat diet. HNG provided experimental consultation and edited the manuscript. DJ provided human NASH specimens and did the clinical grading. GIS supervised metabolic studies. AM designed and supervised the study and provided the direction. NB and AM wrote the manuscript.

## Supplementary Material

Supplemental data

Supplemental Table 3

Supplemental Table 4

Supplemental Table 5

Supplemental Table 6

Supplemental Table 7

Supplemental Table 8

## Version 1 12/02/2021

In-Press Preview

## Version 2 02/01/2022

Electronic publication
