# Supplementary material for: Dyrk1b promotes hepatic lipogenesis by bypassing canonical insulin signaling and directly activating mTORC2 in mice
Source: J Clin Invest. 2022 Feb 1;132(3):e153724. doi: 10.1172/JCI153724 (PMC8803348; doi:10.1172/JCI153724)
Supplement: Supplemental data [file jci-132-153724-s233.pdf]

SFig.1

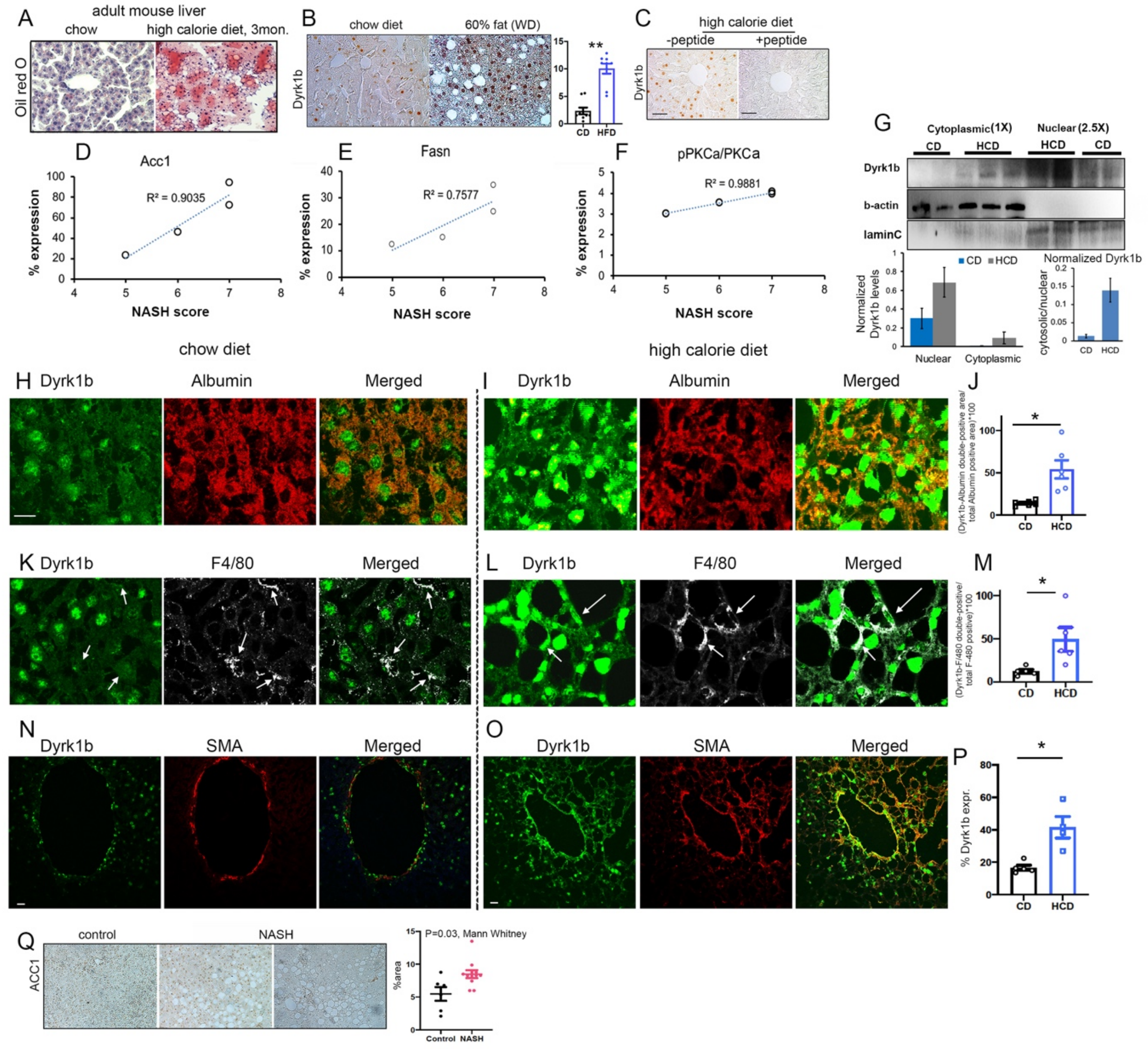

SFig1: Elevated Dyrk1b expression in the liver of mice fed with hcd/hfd and in the biopsies from human patients with NASH. A Lipid accumulation visualized by oil red o staining in the mice fed with a chow diet or a high calorie diet. **B** Dyrk1b expression and quantification in the liver of mice with a standard chow diet or high fat diet (60% calories from fat), n=5 for CD, and n=9 for HFD diet, unpaired t-test, two-tailed. **C** Dyrk1b immunohistochemistry on liver sections with and without preincubation of the anti-Dyrk1b antibody with the corresponding peptide. **D-F** The correlation analysis between expression of Acc1 (D) (R^2=0.9), Fasn (E) (R^2=0.75) and pPKCa/PKCa (F), (R^2=0.98) and NASH severity in the HCD-fed mice, shown in Fig.1B. **G** Dyrk1b expression by WB in nuclear (2.5X) and cytoplasmic (1X) fractions of liver lysates of mice fed with chow diet (CD) or high calorie diet (HCD). n=3 mice each condition. **H-P** Immunofluorescent images and quantification of adult liver of mice fed with CD (H, K, N) or HCD (**I**, L, O), showing co-expression of Dyrk1b with Albumin, F4/80 or SMA as indicated. N=5 mice each condition, unpaired t-test, two-tailed, scale bar= 20µm**. Q** Acc1 expression and quantification in the human control and NASH samples by IHC. p=0.03, Mann Whitney Test.

SFig.2

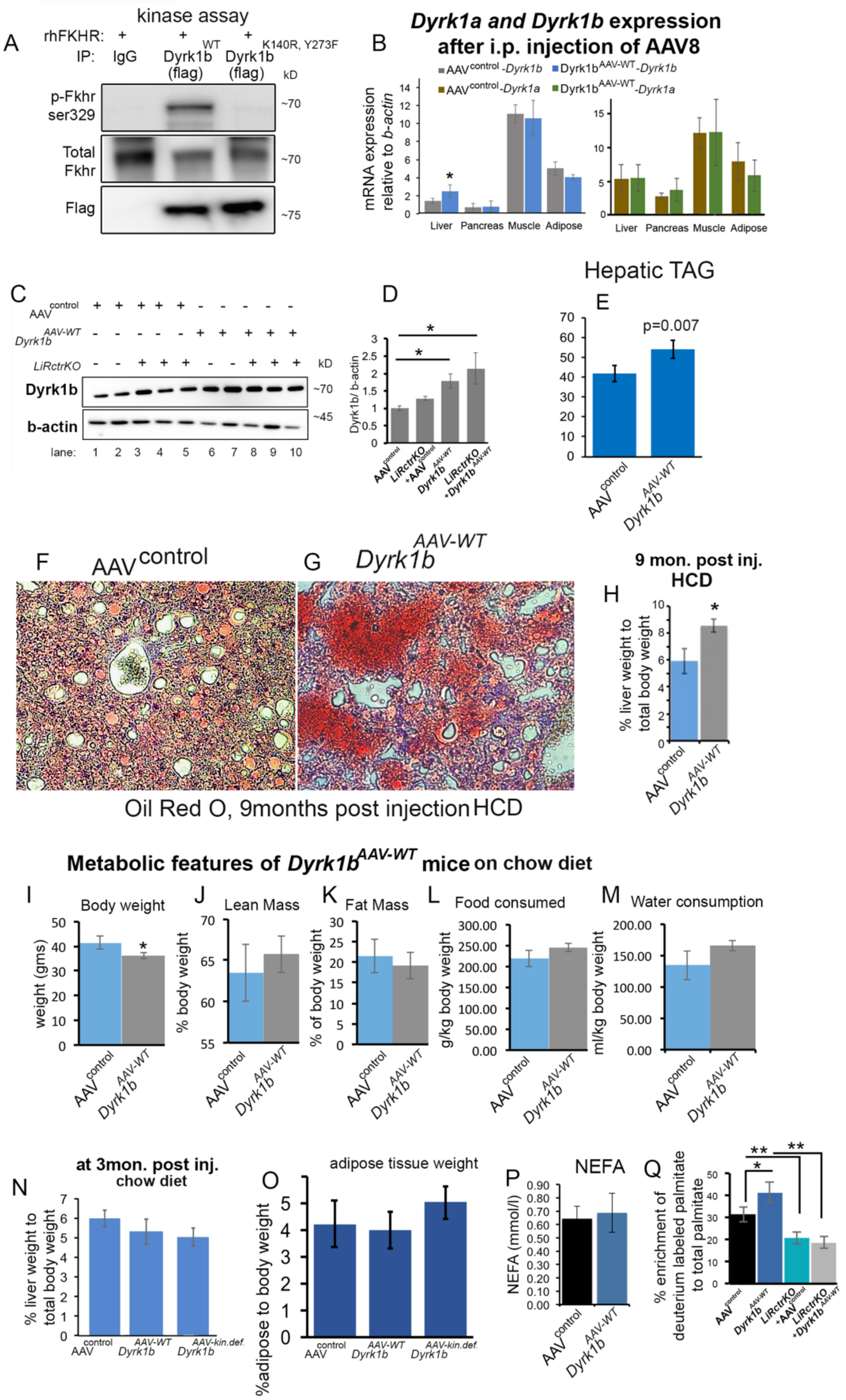

A
kinase assay
rhFKHR: + + +
IP: IgG Dyrk1b WT (flag) Dyrk1b K140R, Y273F (flag)
kD
p-Fkhr ser329
Total Fkhr
Flag
~70
~70
~75
B
Dyrk1a and Dyrk1b expression after i.p. injection of AAV8
AAVcontrol-Dyrk1b
Dyrk1bAAV-WT-Dyrk1b
AAVcontrol-Dyrk1a
Dyrk1bAAV-WT-Dyrk1a
mRNA expression relative to b-actin
Liver Pancreas Muscle Adipose
C
AAVcontrol
Dyrk1bAAV-WT
LiRctrKO
Dyrk1b
b-actin
lane: 1 2 3 4 5 6 7 8 9 10
~70
~45
D
Dyrk1b/ b-actin
AAVcontrol
LiRctrKO +AAVcontrol
Dyrk1bAAV-WT
LiRctrKO +Dyrk1bAAV-WT
E
Hepatic TAG
p=0.007
AAVcontrol
Dyrk1bAAV-WT
F
AAVcontrol
G
Dyrk1bAAV-WT
Oil Red O, 9months post injection HCD
H
9 mon. post inj. HCD
% liver weight to total body weight
Metabolic features of Dyrk1bAAV-WT mice on chow diet
I
Body weight
weight (gms)
J
Lean Mass
% body weight
K
Fat Mass
% of body weight
L
Food consumed
g/kg body weight
M
Water consumption
ml/kg body weight
N
at 3mon. post inj. chow diet
% liver weight to total body weight
AAVcontrol
Dyrk1bAAV-WT
Dyrk1bAAV-kin.def.
O
adipose tissue weight
%adipose to body weight
P
NEFA
NEFA (mmol/l)
Q
% enrichment of deuterium labeled palmitate to total palmitate
AAVcontrol
Dyrk1bAAV-WT
LiRctrKO +AAVcontrol
LiRctrKO +Dyrk1bAAV-WT

SFig2: Metabolic characterization of *Dyrk1b*$^{AAV\text{-}WT}$ mice and validation of kinase-defective Dyrk1b. **A** in-vitro kinase reaction demonstrating that kinase-defective Dyrk1b (K140R, Y273F) fails to phosphorylate its established substrate Fkhr, as assayed by WB. **B** Expression of *Dyrk1b* and *Dyrk1a* in various tissues including liver, pancreas, hindlimb gastrocnemius muscle, epididymal adipose tissue in the AAV^{control} vs. *Dyrk1b*$^{AAV\text{-}WT}$ on chow diet (CD). N>6 mice per group. unpaired t-test, two-tailed. **C, D** Expression and quantification of Dyrk1b by WB in *Dyrk1b*$^{AAV\text{-}WT}$ in the wild type and *LiRictor KO* (liver-specific Rictor knockout) genetic background on chow diet. The littermate controls of LiRctrKO were used. The WB was quantified by ImageJ and One-way ANOVA, Tukey's post hoc test was performed. **E-G** hepatic TAG quantification (E) average±s.e.m (mg TAG/gm protein) littermate controls= 41.85±3.89, *Dyrk1b*$^{AAV\text{-}WT}$ = 54.09± 4.55, p=0.007, unpaired ttest, two-tailed, (F, G) Oil Red O staining in the liver of AAV^{controls} and *Dyrk1b*$^{AAV\text{-}WT}$ subjected to 9 months of high calorie diet, n=4 mice each. **H** Percent liver weight to body weight of mice 9 months on high calorie diet, n=4 mice each, unpaired t-test, two-tailed. **I-M** Several metabolic parameters measured in the indicated mice. The food and water consumption are shown for one representative day. N>5 mice per group. unpaired t-test, Welch corrected, two-tailed. **N** Relative liver weight, % to body weight, in the indicated genotypes at 3months post injection of AAV8 on CD. N>6 mice per group, One-way ANOVA Tukey's post hoc test. **O** Percent of Epidydimal adipose tissue weight to body weight in the indicated mice, N>6 mice per group, One-way ANOVA Tukey's post hoc test. **P** Fasting NEFA (non-esterified free fatty acid) levels in AAV^{control} and *Dyrk1b*$^{AAV\text{-}WT}$ mice on CD, n>6 mice each, unpaired t test, two-tailed. **Q** The percentage enrichment of deuterium in the palmitate: AAV^{control} =31.45 ± 3.56 , *Dyrk1b*$^{AAV\text{-}WT}$ = 42.10 ± 4.7, *LiRctrKO* =20.72 ± 2.81, *LiRctrKO*+ *Dyrk1b*$^{AAV\text{-}WT}$ = 18.48 ± 2.79, n=5 mice in each group. One-way ANOVA, Tukey's posthoc test was performed.*=p<0.05, **=p<0.01, ***=p<0.001.

SFig.3

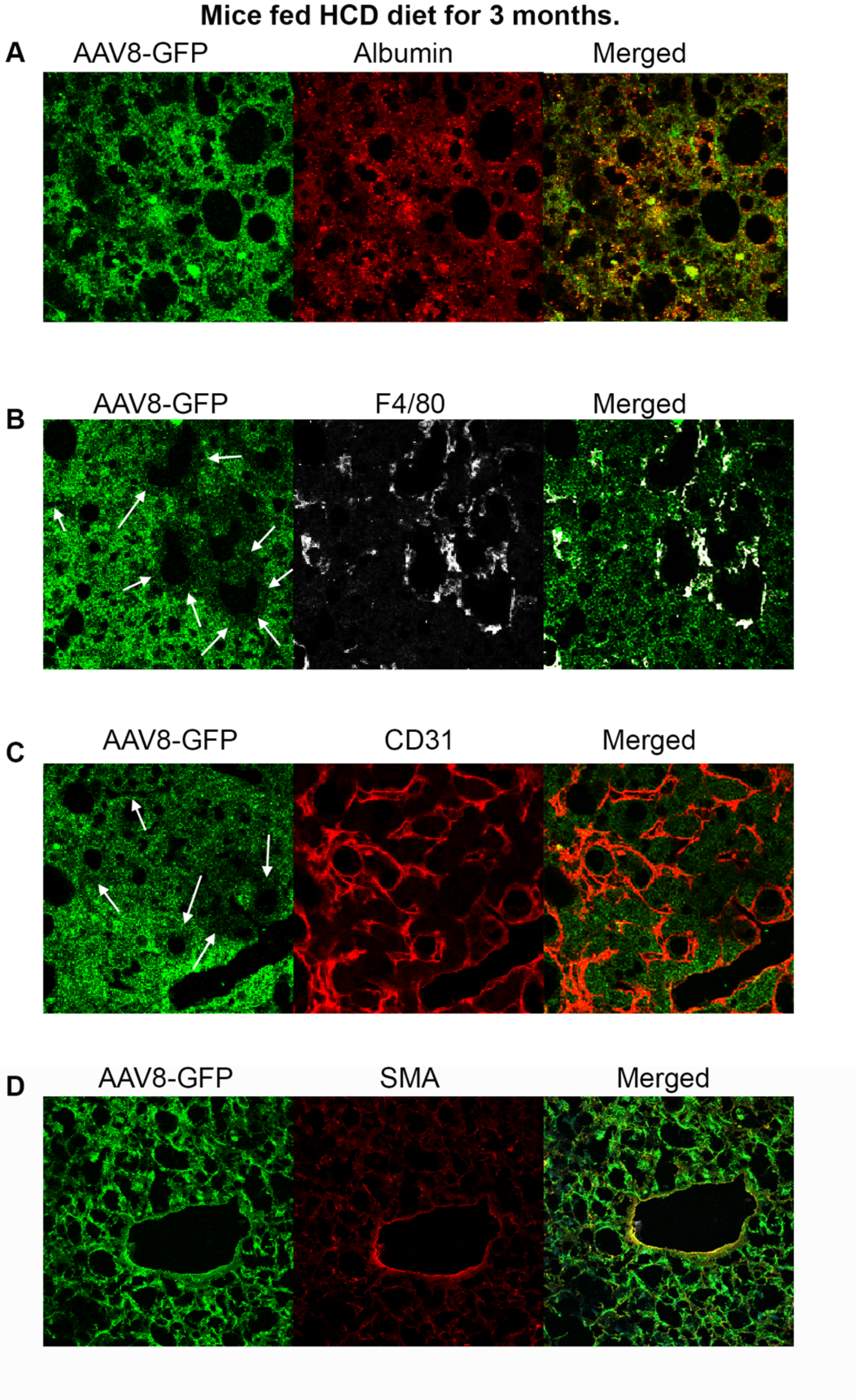

SFig.3: The localization of GFP, transduced by AAV8 with albumin (hepatocyte), macrophages (F4/80), endothelial (CD31) and vascular smooth muscle cells (VSMCs), n>4 mice each.

SFig.4

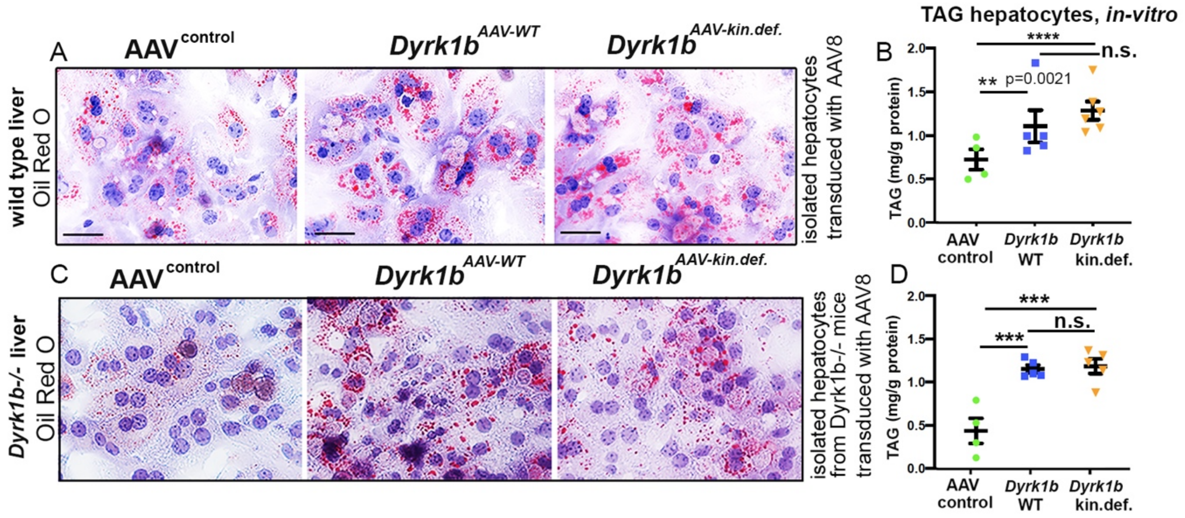

SFig.4: (A-D) Representative pictures of primary hepatocytes from wild type mice (A, B) or *Dyrk1b-/-* mice (C, D) transduced with the designated virus at MOI of 60. The cells were cultured in high glucose, William'sE media along with FBS to stimulate lipid synthesis and fixed in 10% formalin and stained for Oil red O. The hepatic TAG were isolated by Folch method and normalized to total protein levels. (A) TAG (mg)/g protein (average± s.e.m) $AAV^{control}$ = 0.72 ± 0.11, $Dyrk1b^{AAV\text{-}WT}$ = 1.12 ± 0.18 and $Dyrk1b^{AAV\text{-}kin.def}$ =1.28 ± 0.1, *Dyrk1b-/-*= 0.47 ± 0.11, *Dyrk1b-/-* +$Dyrk1b^{AAV\text{-}WT}$ = 1.33 ± 0.044 and $Dyrk1b^{AAV\text{-}kin.def}$ =1.36 ± 0.07, n≥3 technical replicates from n=2 mice each from control and Dyrk1b-/- genotypes. One-way ANOVA, Tukey's test was performed.

SFig.5

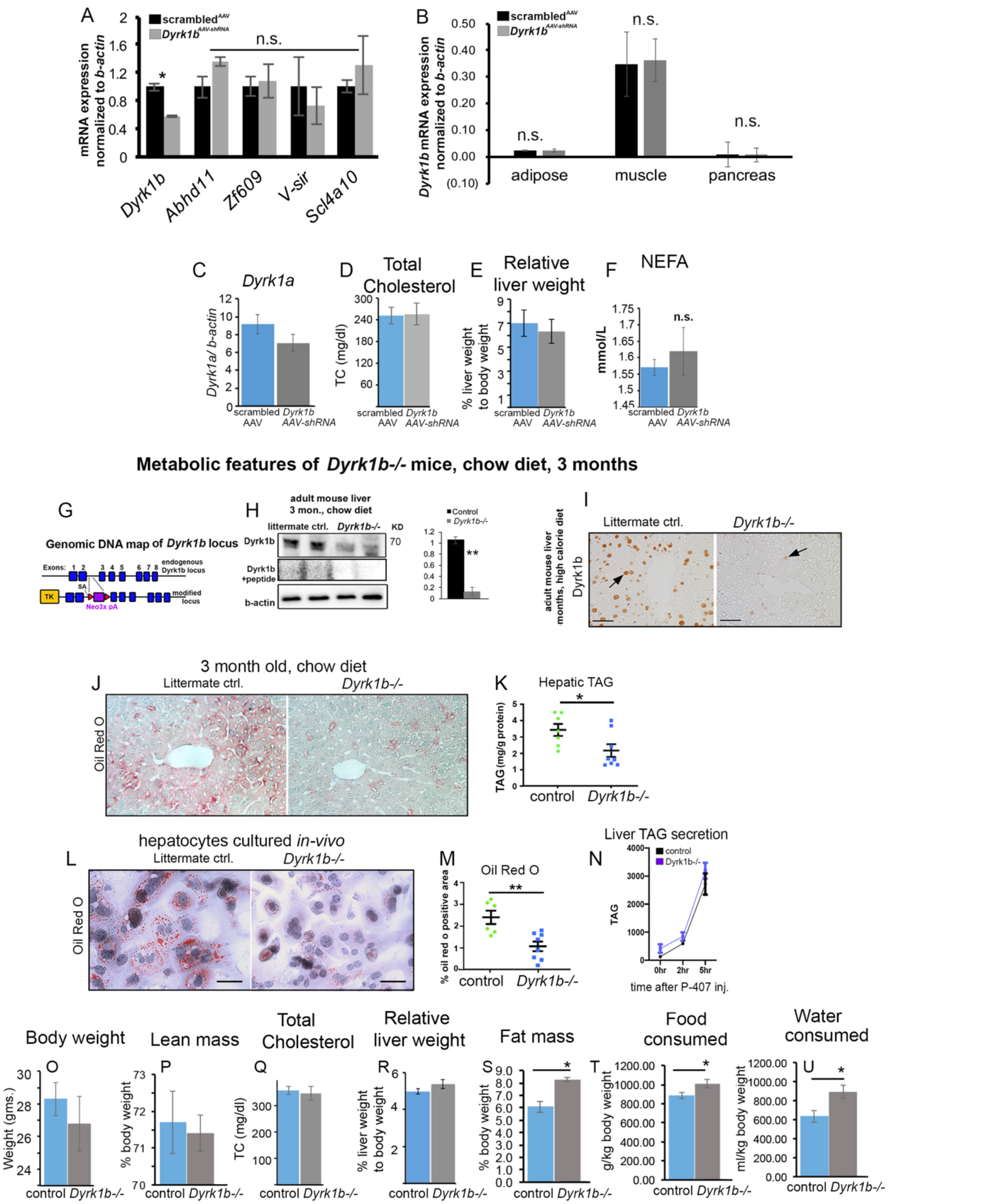

SFig5: Metabolic parameters in *Dyrk1b*$^{AAV\text{-}shRNA}$ and *Dyrk1b-/-* mice.

A mRNA expression of *Dyrk1b* and the predicted off-targets for *Dyrk1b* shRNAs in the mouse liver, fed with HCD. n=5 mice each, unpaired t test, two-tailed**. B** mRNA expression of *Dyrk1b* in epididymal adipose,

gastrocnemius muscle and pancreas in mice injected with AAV8 containing either scrambled shRNA (scrambled^{AAV}) or *Dyrk1b* shRNAs (*Dyrk1b*$^{AAV\text{-}shRNA}$) fed with HCD. **C-F** mRNA expression of *Dyrk1a* (C), total cholesterol (D), relative liver weight (E) and non-esterified fatty acids (F) scrambled^{AAV} and *Dyrk1b*$^{AAV\text{-}shRNA}$ on hcd. N>5 mice per group. unpaired t-test, two-tailed. **G** The endogenous *Dyrk1b* genomic locus (top) and the modified gene-trap (bottom) with a floxed (red arrows) neomycin-polyA gene cassette (purple) preceded by splice acceptor (SA) site. **H** Dyrk1b expression by WB in the liver of littermate controls and *Dyrk1b-/-* mice fed with a standard chow diet. The mice were 3 months old. Each column of WB represents a biological replicate. For lane 2, the antibody was blocked with the corresponding Dyrk1b peptide and the same liver lysates were subjected to the WB with the blocked antibody, unpaired t test, two-tailed. **I** Expression of liver Dyrk1b in *Dyrk1b-/-* mice and the littermate controls on high calorie diet, n=5 mice each group. **J** Oil red O staining on the mouse liver sections of the littermate controls (n=7) and *Dyrk1b-/-* (n=8) mice fed with chow diet. Scale bar= 100µm. **K** Hepatic TAG, isolated by Folch method from snap-frozen liver tissue from littermate controls and *Dyrk1b-/-* mice, normalized to total protein levels on chow diet. average ± s.e.m, TAG (mg) /g protein, littermate controls=3.43 ± 0.30, n=7; *Dyrk1b-/-* = 2.17 ± 0.34, n=8 mice; p=0.028, Mann Whitney test, two-tailed, K-S test=0.02. The mice were fasted for 6 hours prior to sacrifice. **L** Oil Red O staining in the isolated hepatocytes from littermate controls and *Dyrk1b-/-* mice after three-day culture in hepatocyte culture media consisting of Willam'sE + 10%FBS+Supplements. **M** Oil red O quantification by ImageJ for samples shown in panel L. % area (average ± s.d.) controls= 2.4±0.3, n=6; *Dyrk1b-/-*=1.07±0.22, n=8; p=0.0035, unpaired t-test, two-tailed. **N** hepatic TAG secretion at different time intervals after administration of P-407 in the *Dyrk1b-/-* mice vs. littermate controls. N>8 mice per group. **O-U** Several metabolic parameters measured in the *Dyrk1b-/-* mice subjected to metabolic cages for 7 days fed with CD. The food and water consumption are shown for one representative day. N>8 mice per group. unpaired t-test, two-tailed. *p<0.05, **p<0.01, ***p<0.001

SFig.6

***Dyrk1b-/-* mice at 5 months of age, chow diet**

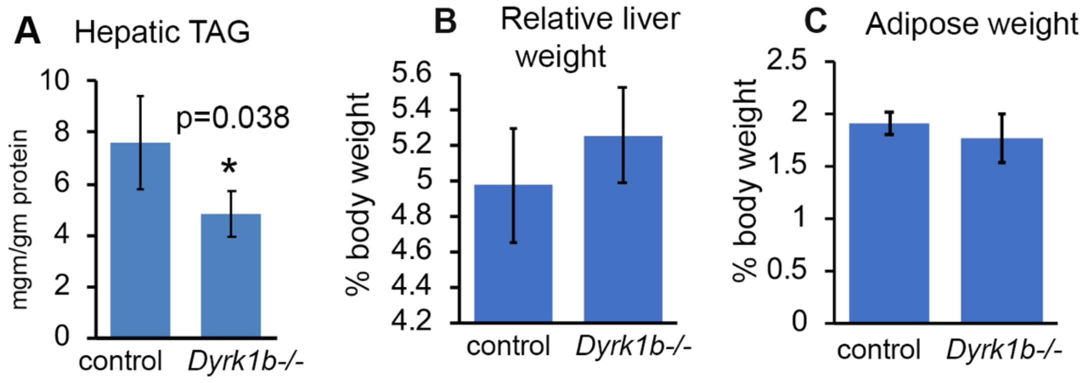

SFig.6: The metabolic parameters in littermate control and *Dyrk1b-/-* mice at 5 months of age. (A) Hepatic TAG, average ± s.e.m, TAG (mg) /g protein, littermate controls=7.60 ± 1.83, n=4; *Dyrk1b-/-* = 4.85± 0.88, n=4 mice; unpaired t test, two-tailed **(B)** percent liver to body weight; **(C)** percent adipose tissue weight of epididymal fat pads are shown.

SFig.7

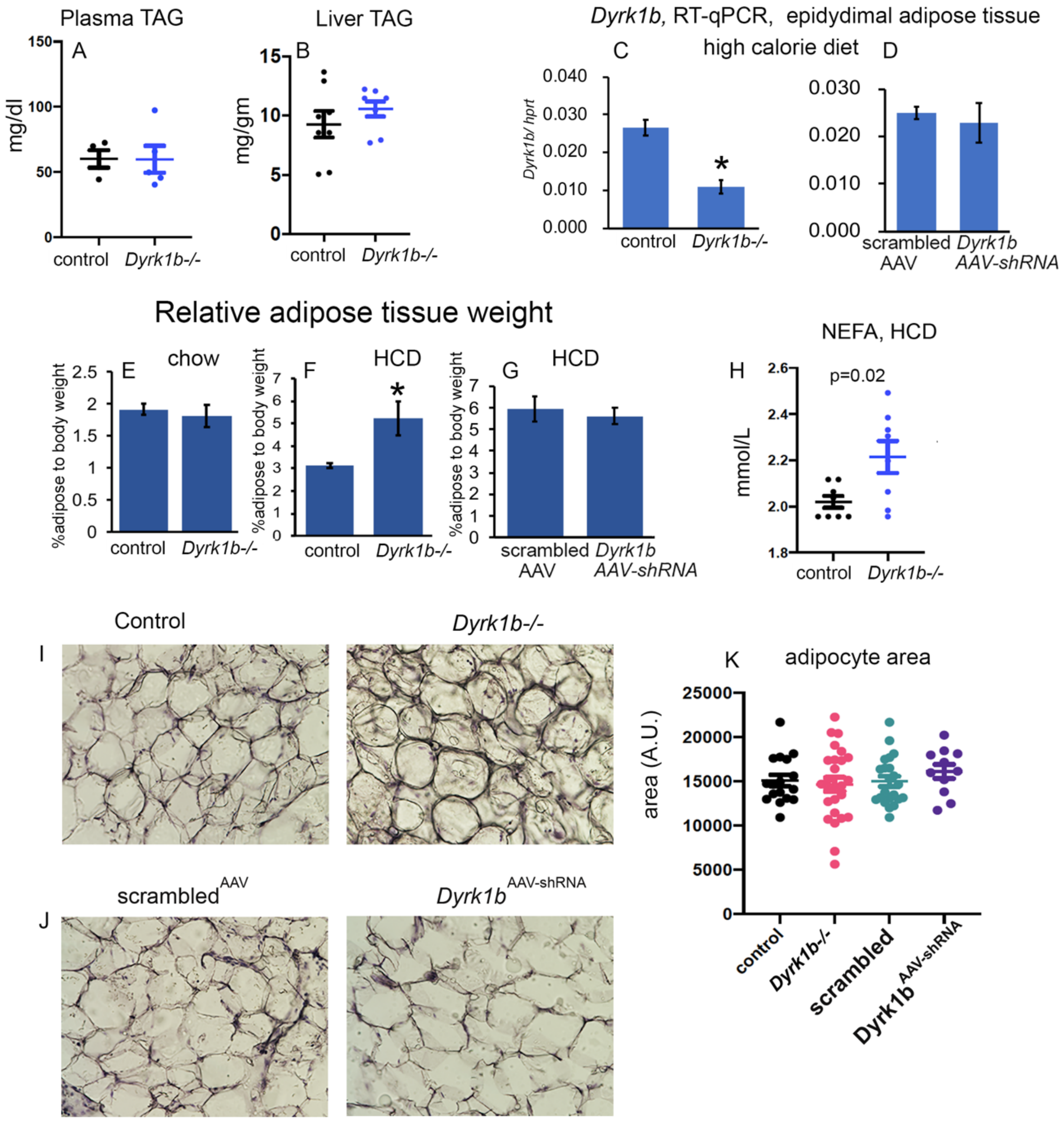

SFig.7: Characterization of *Dyrk1b-/-* on HCD and comparison with *Dyrk1b* $^{AAV\text{-}shRNA}$ mice.
A, B fasting plasma (A) and hepatic TAG (B) in *Dyrk1b-/-* and littermate controls on HCD, n=6 mice each group. **C, D** mRNA expression of *Dyrk1b* in epididymal adipose tissue of *Dyrk1b-/-* mice on HCD (n=6 mice each group) and *Dyrk1b* $^{AAV\text{-}shRNA}$ mice on HCD (n=5 mice each group) relative to their corresponding controls. unpaired t test, two-tailed**. E-G** Percentage adipose tissue weight of epididymal fat pads in the designated mice. n>8 mice each group for E; n=6 mice each for F, n=5 mice each for G. **H** Fasting plasma NEFA in *Dyrk1b-/-* (2.21± 0.09 mmol/L) and littermate controls (2.02±0.03 mmol/L) on HCD. n=6 mice each group, unpaired t test, two-tailed. **I, J** epididymal adipose tissue sections of *Dyrk1b-/-* and *Dyrk1b* $^{AAV\text{-}shRNA}$ mice on HCD. Scale bar=150μm. **K** The cross-sectional area of individual adipocytes in the designated genotypes.

SFig.8

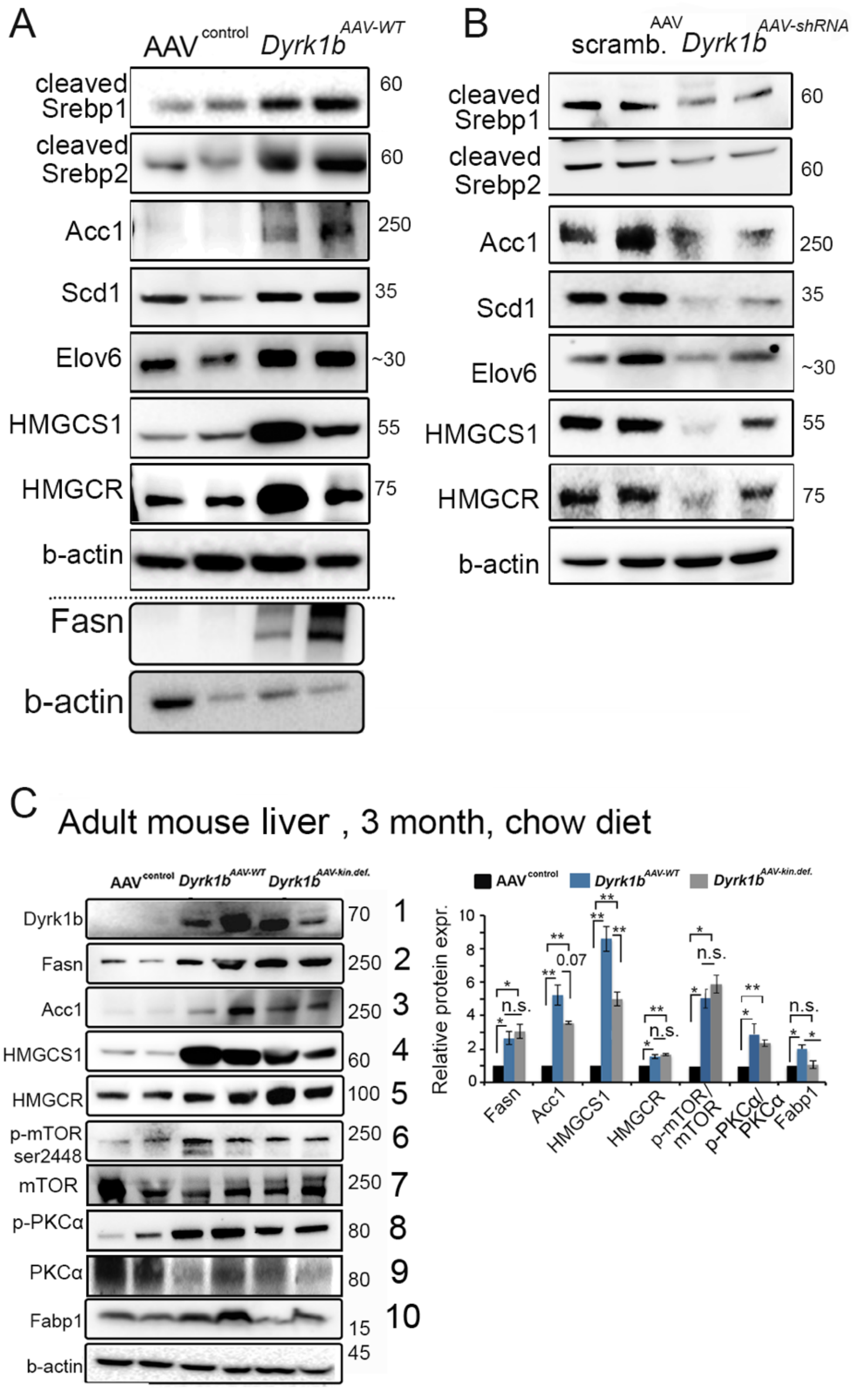

SFig.8: The expression of indicated enzymes in the lipogenic pathway in AAV8 mediated Dyrk1b overexpressing and knockdown mice (A, B), and the kinase deficient *Dyrk1b* (C). The biological replicates in the panel A, B are in addition to the ones shown in Fig.4B, D. One-way ANOVA, Tukey's post hoc test for panel C.

SFig.9

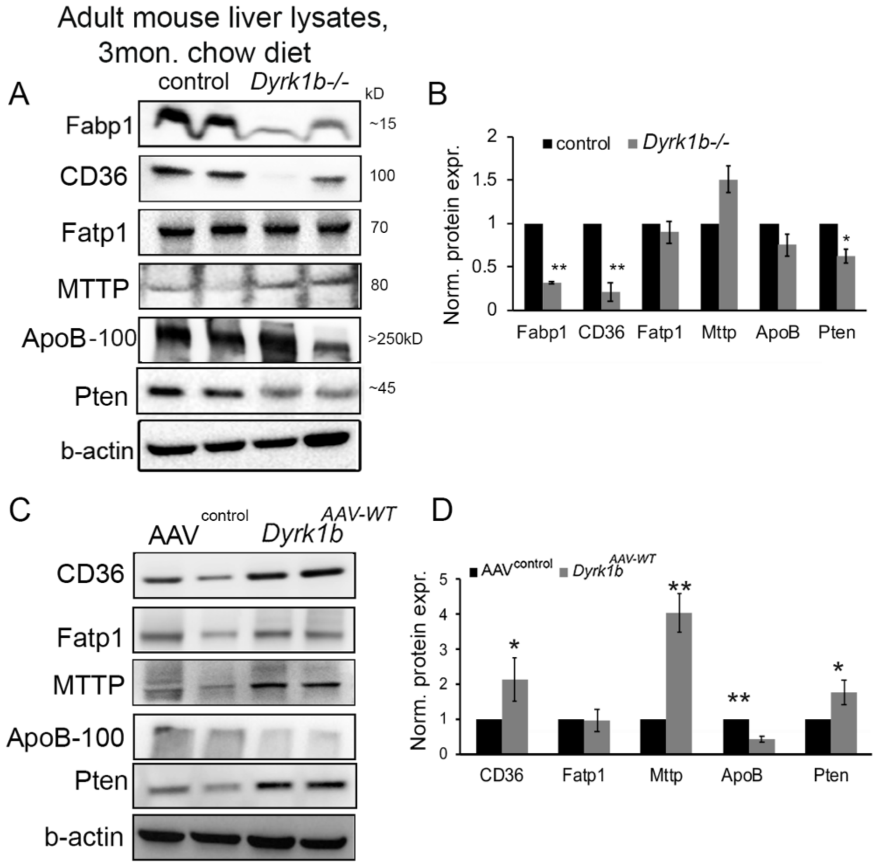

SFig9: Expression and quantification of designated proteins in the *Dyrk1b-/-* (A, B) and *Dyrk1b* $^{AAV-WT}$ (C, D) mice liver assayed by western blot. The WB in panel A were done together with Fig.7B (right panel). unpaired t test, two-tailed.

SFig.10

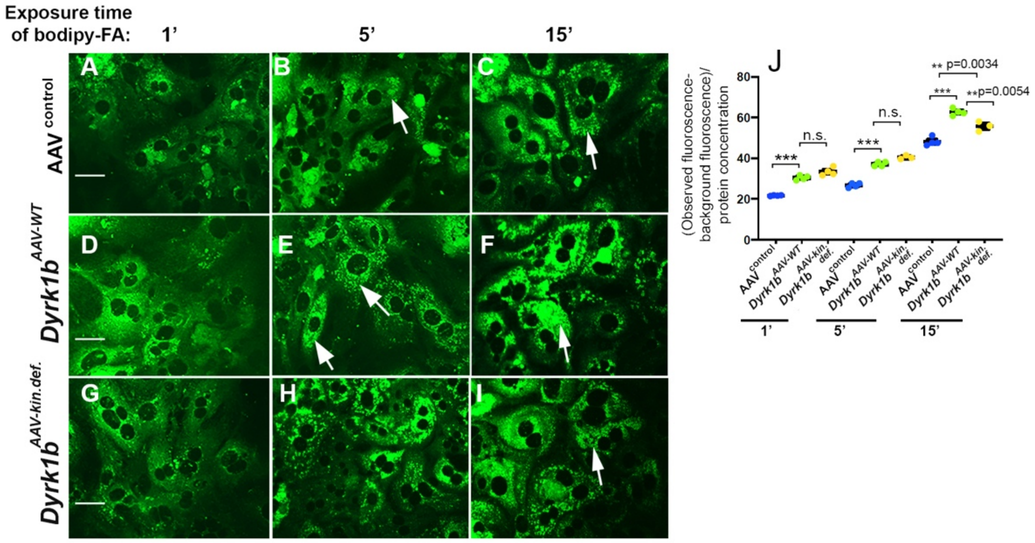

SFig.10: Time-course of Bodipy-FA uptake in wild type hepatocytes transduced with indicated virus. (A-C) wild type hepatocytes transduced with AAV8 containing plasmid with no transgene (AAV control), (D-F) *Dyrk1b*$^{AAV\text{-}WT}$ or (G-I) *Dyrk1b*$^{kin.def.}$ at MOI of 60. The assay was carried out 3 days post infection with AAV8. N=3 biological replicates, and n=4 technical replicates. The cell lysates were quantified for fluorescence. P-values were calculated by One-way ANOVA, Tukey's test.

SFig.11

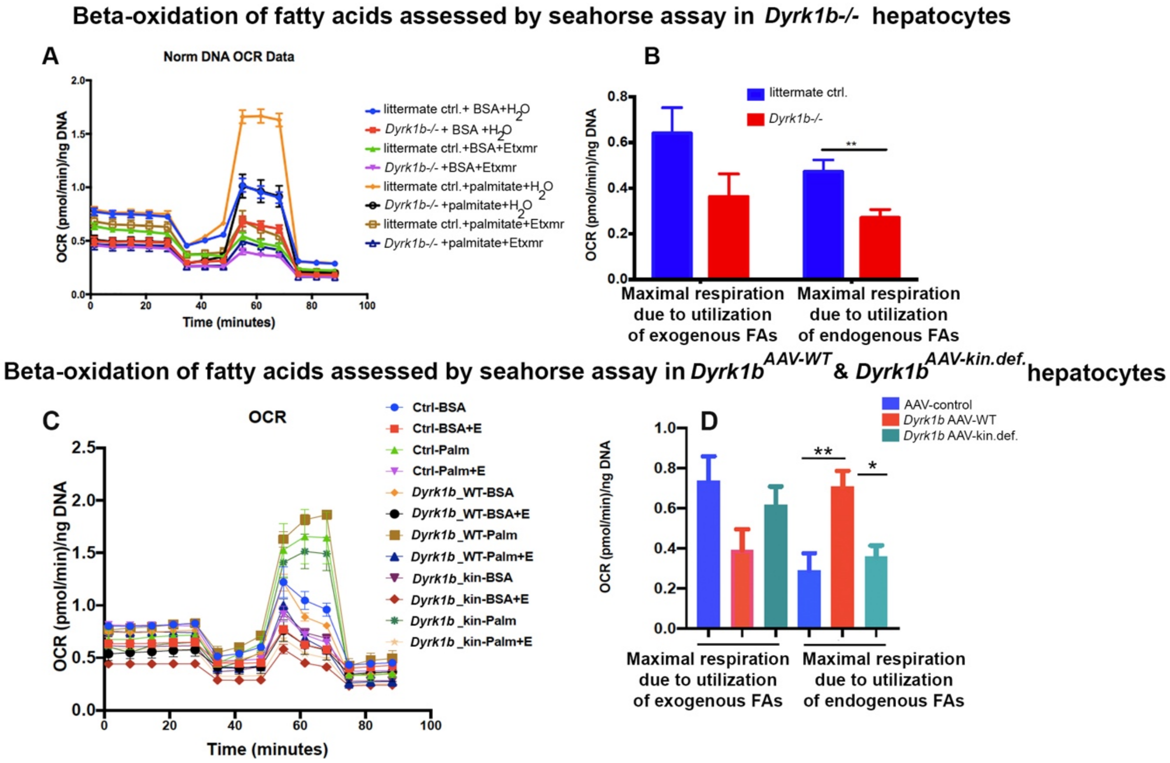

SFig11: Seahorse assay for measuring palmitate oxidation in hepatocytes. The curves show mitochondrial respiration in the presence of BSA or BSA-palmitate, in the presence or absence of Etomoxir, in the hepatocytes from Dyrk1b-/- mice (A,B) and transduced with AAV8 containing empty vector (AAV ^{control}), *Dyrk1b*$^{AAV\text{-}WT}$ or *Dyrk1b*$^{AAV\text{-}kin.def.}$. The oxygen consumption rate (OCR) normalized to total DNA was measured. The hepatocytes were plated in collagen coated dishes, at least 11 replicates were done per condition in a 96 well plate, n=2 biological replicates for each condition and genotype. At the indicated time points, specific reagents for mitochondrial stress test were introduced and OCR was measured. For experimental design, please see materials and methods. unpaired t-test, Welch corrected, two-tailed for panel B, One-way ANOVA Post hoc Tukey's test for panels C, D. *=p<0.05, **=p<0.01, ***=p<0.001.

SFig.12

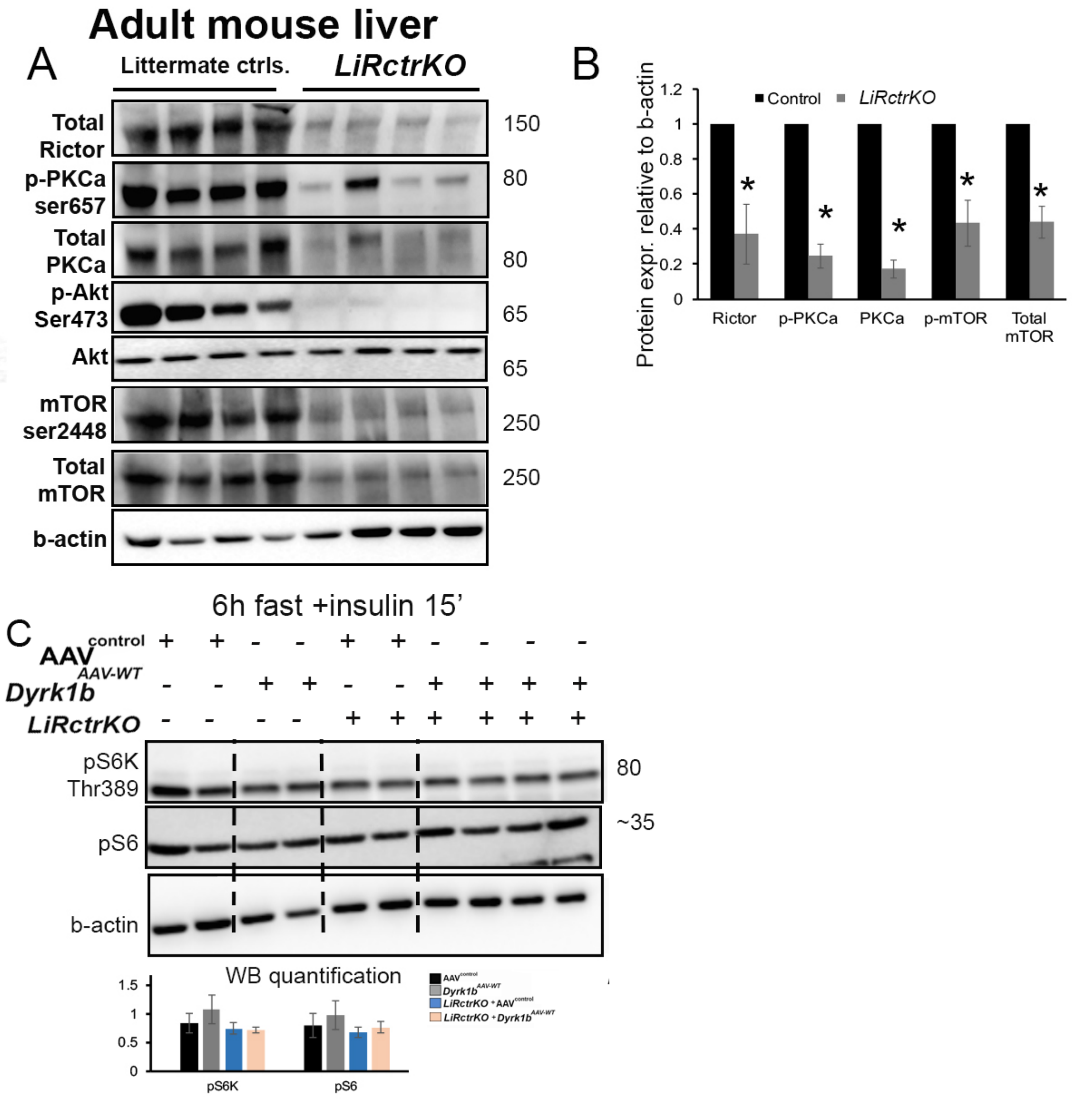

SFig.12: A, B The expression and quantification of proteins indicating the diminished activity of the mTORC2 in the liver-specific *Rictor* knockouts. The liver specific Rictor knockouts were created by *Albumin* promoter driving Cre expression in *the Rictor floxed/floxed* mice. n=4 mice each group, unpaired t test, two-tailed**. C** WB and quantification of mTORC1 readouts in *LiRctrKO* mice liver upon 6h fast followed by 15’ insulin treatment.

SFig.13

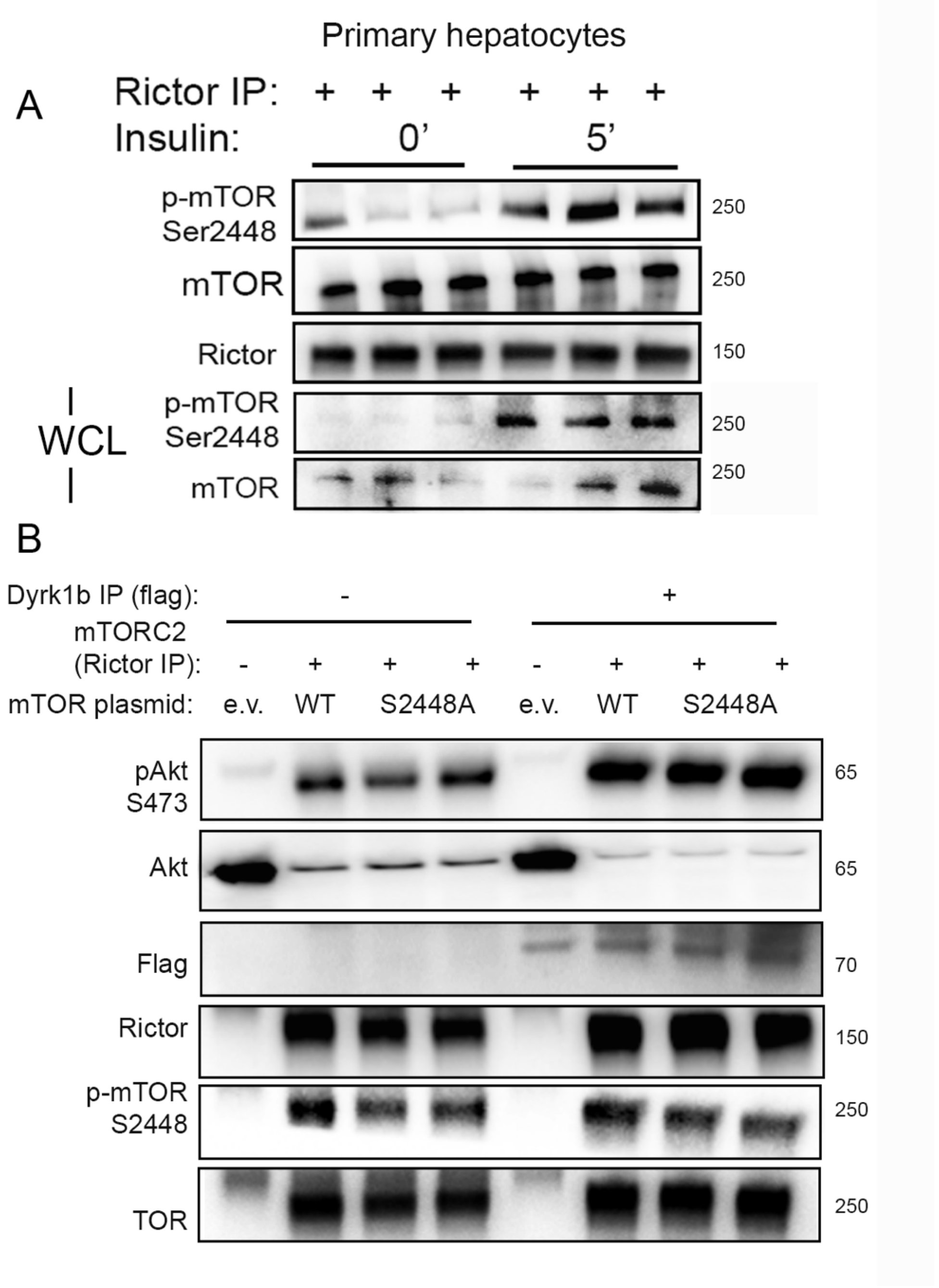

SFig.13: (A) The primary hepatocytes were prepared from wild type C57/B6 mice, serum starved overnight and subjected to insulin for 15', mTORC2 was immunoprecipitated by anti-Rictor antibody and the indicated proteins in the immunoprecipitates were analyzed by WB. WCL: whole cell lysates. n=3 biological replicates for each condition. (B) mTORC2 activity assay carried out between immunoprecipitated Dyrk1b and mTORC2 isolated from HEK cells. mTORC2 was isolated from cells transfected with either empty vector (e.v), wild type mTOR or S2448A mTOR constructs.

SFig.14

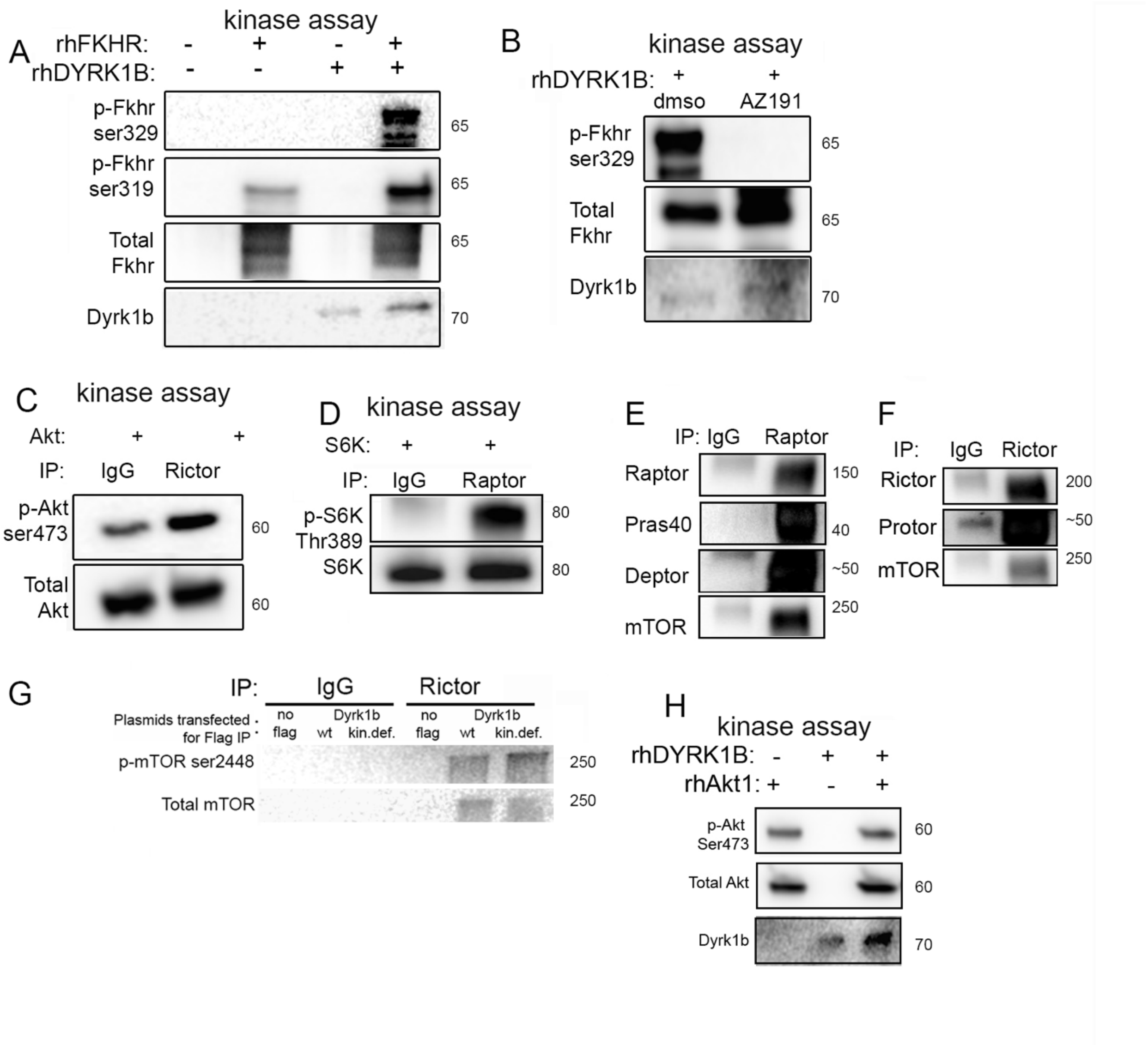

SFig14: Validation of purified rhDYRK1B, pharmacological inhibitor AZ191 and activity of mTOR complexes. A kinase reaction between purified rhDyrk1b and purified rhFkhr to assay for the activity of purified Dyrk1b. **B** kinase assay between purified rhDyrk1b and rhFkhr in the presence of either dmso or AZ191, a kinase inhibitor. AZ191 was pre-incubated with Dyrk1b for 10' before initiating kinase reaction with Fkhr**. C, D** The activity of immunoprecipitated mTORC2 (panel C) (using anti-Rictor antibody) and immunoprecipitated mTORC1 (panel D) (using anti-Raptor antibody, bottom) was assayed by kinase reaction in the presence of purified Akt or purified pS6K. p-Akt Ser473 and p-S6K Thr389 were examined to assay for the activity. **E, F** The subunits of mTORC1 and mTORC2 immunoprecipitated from serum-starved HEK293 cells using anti-Raptor (E) and anti-Rictor antibodies (F). **G** Additional replicates for the kinase assay between mTORC2 and immunoprecipitated Dyrk1b. **H** the kinase reaction between Dyrk1b and Akt to assay for p-Akt Ser473.

SFig.15

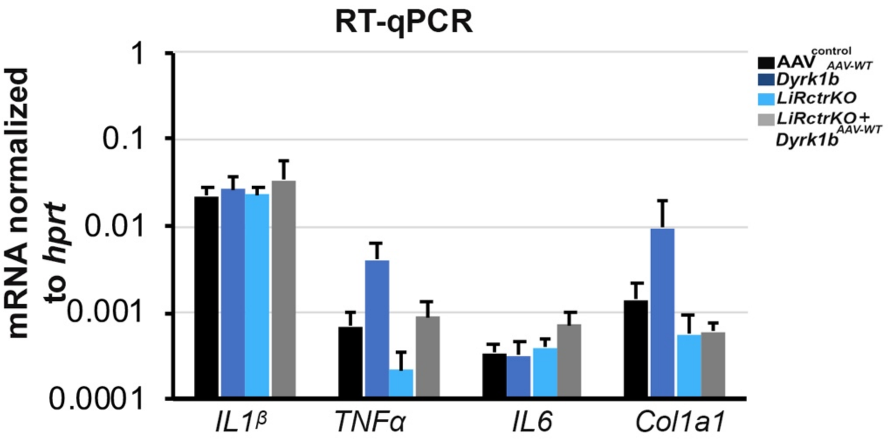

SFig.15: mRNA expression of proinflammatory genes in the liver of designated mice fed with a chow diet. N>5 mice each group, One-way ANOVA, Tukey’s post hoc test.

SFig.16

Plasma ALT levels

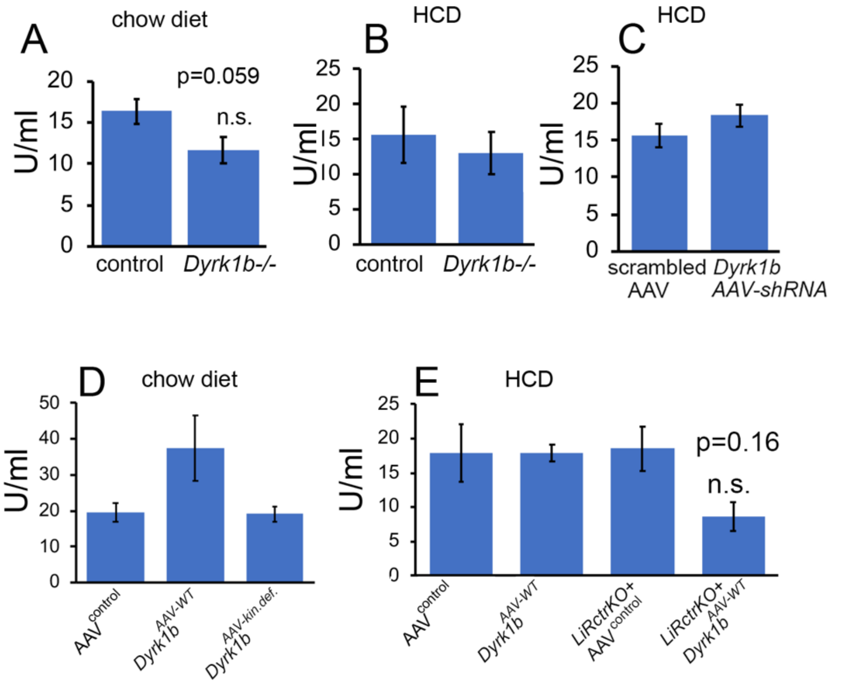

Plasma AST levels

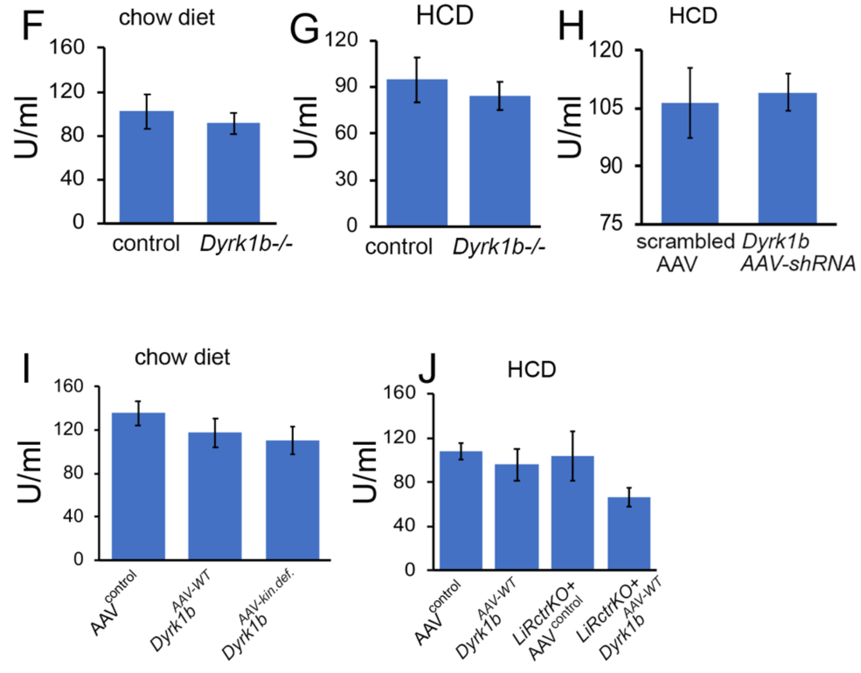

SFig.16: Plasma ALT (A-E) and plasma AST (F-J) in the mice with designated genotypes. n>5 mice each group, One-way ANOVA, Tukey's post hoc test for panels D, E, I, J; for rest of the panels two-sided unpaired t test was performed.

SFig.17

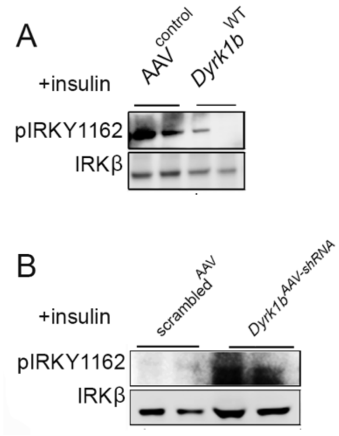

SFig.17: Additional biological replicates for the liver samples from mice with the indicated genotypes.

STable 1: Medical information and percent DYRK1B expression in non-NASH and NASH patients.

STable1

	Non-NASH<br>N= 20	NASH<br>N= 27	p-value
**Age (median ±st dev)**	55 ± 3.52	55 ± 3.13	n.s.
**Gender (M/F)**	8/12	10/17	n.s. chi square
**BMI (kg/m^2)**	26.22 ± 1.47	32.33 ± 0.84	* p=0.022
**Fasting glucose**	118 ± 18.02	142 ± 7.76[A]	n.s., p=0.07
**Triglycerides (mg/dl)**	103.5 ± 10.16	169 ± 17.63	n.s.
**Total Cholesterol (mg/dl)**	147.83 ± 3.98	173.5 ± 11.9	n.s.
**LDL-Cholesterol (mg/dl)**	92.8 ± 7.12	69.4 ± 3.49	n.s.
**HDL-Cholesterol (mg/dl)**	59.4 ± 7.3	44.8 ± 3.05	n.s.
**ALT (U/L)**	27.38 ± 3.26	41.71 ± 3.61	n.s., p=0.07
**AST (U/L)**	29.15 ± 3.9	34.42 ± 2.7	n.s.
**Alkaline Phosphatase**	109.76 ± 11.70	75.85 ± 7.61	n.s.
**Type II Diabetes**	no	12/27[B]	***chi square
**% Dyrk1b positive area**	4.38 ± 0.99	10.08 ± 0.81	***

[A] the patients with type II diabetes were on glucose-lowering medication.
[B] the information on the status of type II diabetes for the other 15 patients was not available.
*p<0.05, Mann Whitney Test was performed for non-parametric distribution.

STable2: The sequences, location, percentage similarity and predicted off-target genes for the four *Dyrk1b* shRNAs and scrambled shRNA.

Target Sequence of *Dyrk1b* shRNA	Location on cDNA and exon	% similarity to *Mus Musculus*, *Dyrk1b*	Predicted off-target; % similarity to *Dyrk1b*
ATCGTGGACTTCGGCAGTTCCTGCCAGCT	Exon6, 1028-1056	100%, Variant 1 to 6	Abhd11; 51%
AAGACCTACAAGCACATCAATGAGGTATA	Exon 3-4, 419-447	100%, Variant 1 to 6	Zpf609; 55%
ACCAGATGAGCCGTATTGTGGAGGTGTTG	Exon8, 1218-1246	100%, Variant 1 to 6	V-Sir; 55%
AACGACAACAGAGCCTACCGATACAGCAA	Exon 10, 1691-1719	100%, Variant 1 to 6	Scl4a10; 51%
Scrambled shRNA: GCACTACCAGAGCTAACTCAGATAGTA		maximum overlap of 4 nucleotides with Dyrk1b	

STable3: Pathways altered in *Dyrk1b*$^{AAV\text{-}WT}$ mouse liver

STable4: Pathways altered in *Dyrk1b-/-* mouse liver

STable5: Pathways related to lipid metabolism altered in *Dyrk1b*$^{AAV\text{-}WT}$ liver

STable6: Pathways related to lipid metabolism altered in *Dyrk1b-/-* liver

STable7: Upstream Regulators of the altered proteome in *Dyrk1b*$^{AAV\text{-}WT}$ liver

STable8: Upstream Regulators of the altered proteome in *Dyrk1b-/-* liver
